# Testing for Genetic Interactions in Complex Disease With Distance Correlation

**DOI:** 10.1002/bimj.70150

**Published:** 2026-09-07

**Authors:** Fernando Castro‐Prado, Javier Costas, Dominic Edelmann, Wenceslao González‐Manteiga, David R. Penas

**Affiliations:** ^1^ Department of Statistics, Faculty of Mathematics University of Santiago de Compostela Santiago de Compostela, Galicia Spain; ^2^ Psychiatric Genetics Laboratory, Santiago Health Research Institute (IDIS) University Hospital Santiago de Compostela, Galicia Spain; ^3^ Biostatistics Department German Cancer Research Center (DKFZ) Heidelberg, Baden‐Württemberg Germany; ^4^ Computational Biology Laboratory Spanish National Research Council (MBG‐CSIC) Pontevedra, Galicia Spain

**Keywords:** association measures, distance correlation, epistasis, genomics of complex diseases, schizophrenia

## Abstract

Understanding epistasis (genetic interaction) may shed some light on the genomic basis of common diseases, including disorders of maximum interest due to their high socioeconomic burden, like schizophrenia. Distance correlation is an association measure that characterizes general statistical independence between random variables, not only the linear one. Here, we propose distance correlation as a novel tool for the detection of epistasis from case‐control data of single‐nucleotide polymorphisms. On the methodological side, we highlight the derivation of the explicit asymptotic null distribution of the test statistic. We show that this is the only way to obtain enough computational speed for the method to be used in practice, in a scenario where the resampling techniques found in the literature are impractical. Our simulations show satisfactory calibration of significance, as well as comparable or better power than existing methodology. We conclude with the application of our technique to a schizophrenia genetics dataset, obtaining biologically sound insights.

## 1 Introduction

The role of heredity in psychiatry has been studied for almost a century, with Pearson (1931) not having “the least hesitation” in asserting its relevance. Today, it is known that a majority of psychiatric disorders are multifactorial, complex traits. They occur as a result of a combination of genetic and environmental factors, none of which are necessary or sufficient. Furthermore, the individual effect of each of them is generally trifling. More precisely, the genome can explain up to 80% of the susceptibility to some of these diseases, like schizophrenia (Sullivan et al. 2018).

The genetic susceptibility to a psychiatric disorder is spread across a large number of variants along the genome. Although the specialized literature usually focuses simply on additive models (International Schizophrenia Consortium 2009), biological knowledge suggests that gene–gene interactions (or *epistasis*) could be one of the factors that explain the phenomenon of *missing heritability*, which contributes to the inefficiency of genome‐wide association studies (GWAS) when it comes to explaining causality of complex diseases (Brandes et al. 2022; Manolio et al. 2009). Evidence from studies on model organisms also supports the importance of genetic interactions in the understanding of complex traits (Mackay and Moore 2014).

We are interested in datasets of case‐control GWASs (i.e., a collection of genotypes of “healthy” individuals and “patients”) for schizophrenia. The statistical challenge hinges on using these data to detect pairs of genetic variants that significantly increase or decrease the susceptibility to develop schizophrenia, which further research can confirm with biological criteria.

These data correspond to *single‐nucleotide polymorphisms* (SNPs), which are variants on one of the “letters” of the DNA (i.e., each of them occurs at one specific point of the genome). Given that we will only consider autosomal variants, each individual can carry 0, 1, or 2 copies of the *minor* allele (the least frequent of the two variants) in their diploid genome. This setting requires performing statistical inference in a context of high dimension and low sample size, where the covariates are *ternary* (discrete with support of cardinality 3).

Supporting Information Appendix A reviews the extremely wide variety of approaches to the detection of epistasis that can be found in the biostatistical literature. The main conclusion of that study effort is that there is no clear winner among the different available techniques, which justifies the maintained interest in this problem over the past few years.

In Table S1, it is shown that one conspicuous epistasis detector (Kam‐Thong et al. 2011) is based on scanning for differential behaviors of (Pearson's) correlations between cases and controls. This is unsurprising, since several authors (Camacho et al. 2005; De la Fuente 2010; D'Haeseleer et al. 2000) support the idea of correlation tests in this context when the data are continuous (gene expression, metabolomics, and so forth), which, however, is not the case with SNPs (ternary variables).

Moreover, such techniques usually rely on the normality of the covariates, a hypothesis that might be excessively restrictive in some cases. Therefore, the procedure by Cai and Liu (2016) contains an interesting approach, as they manage to establish a rigorous theoretical framework for the kind of correlation tests that are convenient for epistasis detection. This technique is part of the hot topic of hypothesis testing on high‐dimensional covariance structures, which has been developed almost from scratch during the past few years (Cai 2017). A short technical introduction to large‐scale correlation tests (LCTs) can be found in Supporting Information Appendix C.

**FIGURE 1:**
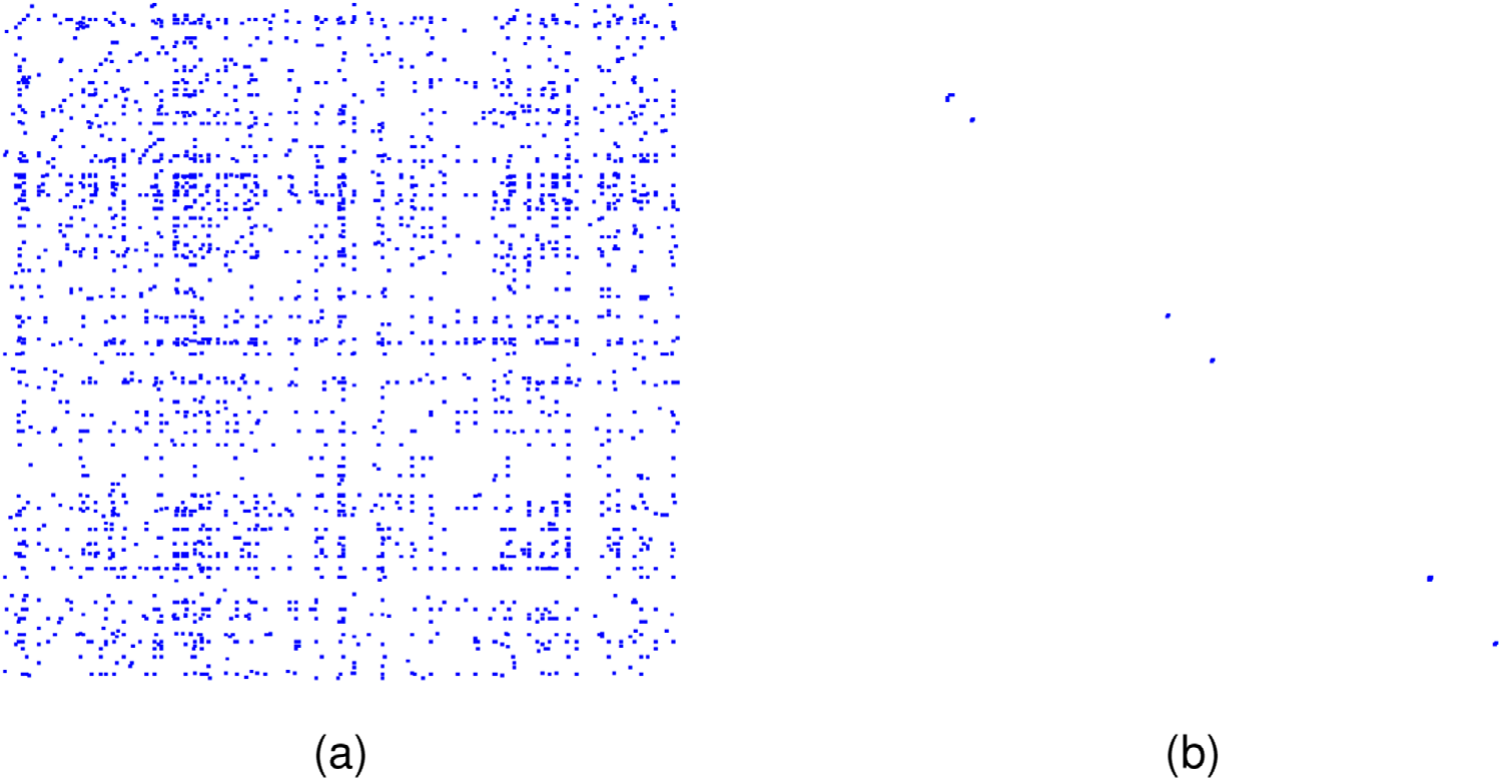
Adjacency matrix of the putative epistatic network detected by the LCT method encoded as “LCT‐B” for (a) gene expression data for prostate cancer (Broad Institute) and (b) SNP data for schizophrenia (Health Research Institute, Santiago de Compostela, Spain).

We have implemented the LCTs of Cai and Liu (2016) in the *R* programming language to further illustrate the motivation of our work. We first reproduced the real‐data example from the original LCT article, obtaining the adjacency matrix in Figure 1a. To accomplish that, we applied the method known as LCT‐B to the data by Singh et al. (2002), in which dimensionality was trimmed down to 500 using the Welch–Satterthwaite test (Behrens–Fisher problem). Since the variables involved are assumed to be continuous, the LCT‐B yields believable results, in the sense that the resulting matrix is sparse, but not too much. However, a biological validation of all those results would be extremely difficult to carry out.

On the other hand, when the schizophrenia SNP data (remarkably discrete) are analyzed, the adjacency matrix looks very different (Figure 1b) from the previous one (Figure 1a). The only nonzero elements are close to the diagonal, owing to the fact that the only pairs that are being detected are in linkage disequilibrium (i.e., the frequency of such SNP pairs is significantly different from the product of the marginal frequencies, due to physical proximity within a certain chromosome). Such findings are useless from the point of view of psychiatric genetics because they do not show an association that is related to schizophrenia, but rather one that is independent of this disorder.

Some authors, like Kam‐Thong et al. (2011), argue that treating clearly discrete SNP data as continuous is an acceptable simplification. Nevertheless, even if that could be anecdotally true in some specific setting, this is clearly not the case, as Figure 1b clearly displays.

The unsatisfactory behavior of the LCT‐B, one of the most robust techniques for epistasis detection (when applied to a different setting from the one it was originally intended for), is the main motivation of the present article. In this context, it is justified to wonder which association measures characterize the independence of ternary variables, as well as how to extend the LCTs by Cai and Liu (2016) to less stringent conditions so that they become applicable to SNP data.

The remainder of the paper is organized as follows. Section 2 summarizes the state of the art in the characterization of general statistical independence in metric spaces, focusing on an association measure named *distance correlation*. Section 3 introduces a novel testing procedure for independence in ternary data. Some results of our simulation study are reported in Section 4. In Section 5, we apply the method to a genomic dataset of schizophrenia. Concluding remarks are given in Section 6.

## 2 Distance Correlation in Metric Spaces

The *energy of data* (Székely and Rizzo 2017) is a branch of mathematical statistics that has been recently developed, and it includes the characterization of statistical independence in Euclidean spaces via an association measure known as *distance correlation*.

The extension of distance correlation to metric spaces is a nontrivial issue, which will be concisely summarized hereinafter. For a more detailed review of this theoretical framework, please refer to Castro‐Prado and González‐Manteiga (2020).

### 2.1 Distance Correlation in Euclidean Spaces

When two random elements (vectors) X and Y are Euclidean‐space‐valued (let X be L‐dimensional and Y be M‐dimensional, for $L,M\in Z^{+}$), it is possible to define an association measure that characterizes their independence with generality, which is called *distance correlation* (Székely et al. 2007). First, distance covariance should be defined, as a weighted $L^{2}$ norm of the difference of the joint characteristic function and the product of the marginals:

$$\begin{array}{ccc} & & \mathrm{dCov}\left(X,Y\right):={\left\|\varphi_{X,Y}-\varphi_{X}\varphi_{Y}\right\|}_{w}\\ & & \;\equiv \sqrt{\int_{R^{L}\times R^{M}}{\left|\varphi_{X,Y}\left(t,s\right)-\varphi_{X}\left(t\right)\varphi_{Y}\left(s\right)\right|}^{2}w\left(t,s\right)\;dt\;ds}; \end{array}$$

where w is a weight function which is dependent on the dimension of the Euclidean spaces in which the supports of X and Y are contained. As usually:

$$\varphi_{X}\left(t\right):=E\left[\exp\left(i\langle t,X\rangle \right)\right],\;t\in R^{L}.$$

Logically, distance correlation is defined as the quotient of covariance and the product of standard deviations (as long as none of the latter vanish):

$$\mathrm{dCor}\left(X,Y\right):=\frac{\mathrm{dCov}(X,Y)}{\sqrt{\mathrm{dCov}(X,X)\mathrm{dCov}(Y,Y)}},$$

and so it has no sign. It is an improved version of Pearson's correlation squared because it has values in [0,1] and, more importantly, it is zero if *and only if* X and Y are (statistically) independent.

However convoluted the initial definition of *dCor* is, the sample version can easily be computed. Given a paired sample $\left(X_{1},Y_{1}\right),\text{…},\left(X_{n},Y_{n}\right)\;\text{IID}\;\left(X,Y\right);$ let $a_{ij}:=d\left(X_{i},X_{j}\right)$ be the Euclidean distance between the Xs with indices i,j∈{1,…,n}. Then, the doubly centered distances are

(1) $$A_{ij}:=a_{ij}-\frac{1}{n}\sum_{k=1}^{n}a_{ik}-\frac{1}{n}\sum_{k=1}^{n}a_{kj}+\frac{1}{n^{2}}\sum_{k,l=1}^{n}a_{kl}\;.$$

If ${\left\{b_{ij}\right\}}_{i,j}$ and ${\left\{B_{ij}\right\}}_{i,j}$ are analogously defined for ${\left\{Y_{i}\right\}}_{i}$, the empirical distance covariance is the nonnegative square root of

(2) $${\hat{\mathrm{dCov}}}_{n}{\left(X,Y\right)}^{2}:=\frac{1}{n^{2}}\sum_{i,j=1}^{n}A_{ij}B_{ij}\;.$$

The above estimator is reminiscent of the following alternative representation of ${\mathrm{dCov}}^{2}$:

$$\begin{array}{cc} & \mathrm{dCov}{\left(X,Y\right)}^{2}\\ & \;=E\left[\left(d\left(X,X^{\prime }\right)-E\left\{d\left(X,X^{\prime \prime }\right)\right\}-E\left\{d\left(X^{\prime },X^{\prime \prime \prime }\right)\right\}+E\left\{d\left(X^{\prime \prime },X^{\prime \prime \prime }\right)\right\}\right)\right.\\ & \;\left.\times \;\left(d\left(Y,Y^{\prime }\right)-E\left\{d\left(Y,Y^{\prime \prime \prime \prime }\right)\right\}-E\left\{d\left(Y^{\prime },Y^{\prime \prime \prime \prime \prime }\right)\right\}+E\left\{d\left(Y^{\prime \prime \prime \prime },Y^{\prime \prime \prime \prime \prime }\right)\right\}\right)\right], \end{array}$$

which is valid as long as moments of order 2 are finite (Jakobsen 2017, Remark 4.6). In the above equation, primed letters refer to independent and identically distributed (IID) copies of the corresponding random vector, and *d* denotes the Euclidean distance.

Whenever {X,Y} are independent and have finite first moments, the asymptotic null distribution of a scaled version of the preceding statistic is a linear combination of independent chi‐squared variables with one degree of freedom. More precisely,

$$n\;{\hat{\mathrm{dCov}}}_{n}{\left(X,Y\right)}^{2}\overset{D}{\underset{n\to \infty }{⟶}}\sum_{j=1}^{\infty }\lambda_{j}Z_{j}^{2},$$

where ${\left\{Z_{j}\right\}}_{j}$ are IID N(0,1) and where ${\left\{\lambda_{j}\right\}}_{j}\subset R^{+}$.

Unfortunately, knowing the form of the theoretical null distribution is often not helpful in practice. As a result, all the distance correlation literature we are aware of resorts to resampling techniques when it comes to approximating the critical values for the independence test. They generally design the resampling scheme based on the information that $H_{0}$ provides, which in this case (i.e., independence) leads to permutation testing. The theoretical quadratic form is not used, due to the difficulty of estimating the $\lambda_{j}s$, since one would need to deal with an abstract linear operator and obtain its nonzero eigenvalues (potentially, an infinity of them), all this under no model assumptions (note that our setting is nonparametric); see, for example, Jakobsen (2017) or Székely et al. (2007).

### 2.2 The Generalized Distance Covariance

Let θ be a (Borel) probability distribution on X×Y, where $\left(X,d_{X}\right)$ and $\left(Y,d_{Y}\right)$ are separable metric spaces. Its distance covariance is defined as

$$\mathrm{dcov}\left(\theta \right):=\int_{{\left(X\times Y\right)}^{2}}d_{\mu }\left(x,x^{\prime }\right)d_{\nu }\left(y,y^{\prime }\right)\;d\theta^{2}\left(\left(x,y\right),\left(x^{\prime },y^{\prime }\right)\right),$$

where (μ,ν) are the marginals of θ on (X,Y) and are assumed to both have finite first moments (Jakobsen 2017; Lyons 2013). Functions $d_{\mu }$ and $d_{\nu }$ are the doubly centered versions of $d_{X}$ and $d_{Y}$, respectively:

$$\begin{array}{cc} d_{\mu }:\; & X\times X\;⟶R\\ & \left(x_{1},x_{2}\right)\mapsto d_{X}\left(x_{1},x_{2}\right)-a_{\mu }\left(x_{1}\right)-a_{\mu }\left(x_{2}\right)+D\left(\mu \right); \end{array}$$

where

$$\begin{array}{cc} a_{\mu }:\; & X⟶R\\ & x\mapsto \int_{X}d_{X}\left(x,x^{\prime }\right)\;d\mu \left(x^{\prime }\right); \end{array}$$

$$D\left(\mu \right):=\int_{X}a_{\mu }\;d\mu =\int_{X^{2}}d_{X}\;d\mu \times \mu .$$

Like ordinary covariance, *dcov* vanishes under independence as a result of Fubini's theorem:

dcov(μ×ν)={D(μ)−2D(μ)+D(μ)}{D(ν)−2D(ν)+D(ν)}=0.

### 2.3 Distance Covariance in Negative‐Type Spaces

The fact that

θ=μ×ν⇒dcov(θ)=0

makes it natural to wonder which spaces ensure that the reciprocal implication also holds. The answer is *strong negative‐type* spaces, since in them dcov(θ) can be presented as an injective function of θ−μ×ν.

A metric space $\left(X,d_{X}\right)$ is said to be of negative type if and only if

$$\begin{array}{ccc} & & \forall n\in Z^{+};\;\forall x,y\in X^{n}:\\ & & \;2\sum_{i,j=1}^{n}d_{X}\left(x_{i},y_{j}\right)\geq \sum_{i,j=1}^{n}\left\{d_{X}\left(x_{i},x_{j}\right)+d_{X}\left(y_{i},y_{j}\right)\right\}. \end{array}$$

There are many familiar examples of negative‐type spaces, including all Hilbert spaces (and therefore, all Euclidean geometries).

If $\left(X,d_{X}\right)$ has negative type, the inequality $D(\mu_{1}-\mu_{2})\leq 0$ holds for any probability distributions $\mu_{1},\mu_{2}$ on X with finite first moments. On top of that, if the operator D separates probability measures (with finite first moments) in $\left(X,d_{X}\right)$, that space is said to have *strong* negative type:

$$D\left(\mu_{1}-\mu_{2}\right)=0\Leftrightarrow \mu_{1}=\mu_{2}.$$

Whenever X and Y have strong negative type,

$$\mathrm{dcov}\left(X,Y\right)\overset{\text{def.}}{=}\mathrm{dcov}\left(\theta \right)=0\Leftrightarrow \;X,Y\;\text{independent,}$$

for any random element (X,Y)∼θ with values in X×Y.

### 2.4 Nonparametric Test of Independence in Metric Spaces

For $n\in Z^{+}$, the *empirical measure* associated with a certain sample, ${\left\{\left(X_{i},Y_{i}\right)\right\}}_{i=1}^{n}\;\text{IID}\;\left(X,Y\right)∼\theta$, is defined as customarily:

$$\theta_{n}:=\frac{1}{n}\sum_{i=1}^{n}\delta_{\left(X_{i},Y_{i}\right)},$$

where $\delta_{z}$ denotes point mass at z∈X×Y.

It is easy to see that the natural estimator $\mathrm{dcov}(\theta_{n})$ is a V‐statistic

$$\mathrm{dcov}\left(\theta_{n}\right)=\frac{1}{n^{6}}\sum_{i_{1}=1}^{n}\cdots \sum_{i_{6}=1}^{n}h\left({\left(X_{i_{\lambda }},Y_{i_{\lambda }}\right)}_{\lambda =1}^{6}\right),$$

whose (nonsymmetric) kernel h is given by

$$\begin{array}{cccc} h:\; & {\left(X\times Y\right)}^{6} & ⟶ & R\\ & {\left((x_{i},y_{i})\right)}_{i=1}^{6} & \mapsto & \left\{d_{X}\left(x_{1},x_{2}\right)+d_{X}\left(x_{3},x_{4}\right)-d_{X}\left(x_{1},x_{3}\right)-d_{X}\left(x_{2},x_{4}\right)\right\}\\ & & & \times \left\{d_{Y}\left(y_{1},y_{2}\right)+d_{Y}\left(y_{5},y_{6}\right)-d_{Y}\left(y_{1},y_{5}\right)-d_{Y}\left(y_{2},y_{6}\right)\right\}. \end{array}$$

If θ is the product of its marginals and these are nondegenerate, the asymptotic null distribution of the V‐statistic is

$$n\mathrm{dcov}\left(\theta_{n}\right)\overset{D}{\underset{n\to \infty }{⟶}}\sum_{i=1}^{\infty }\lambda_{i}\left(Z_{i}^{2}-1\right)+D\left(\mu \right)D\left(\nu \right),$$

where ${\left\{Z_{i}\right\}}_{i\in Z^{+}}\;\text{IID}\;N\left(0,1\right)$ and where ${\left\{\lambda_{i}\right\}}_{i\in Z^{+}}\subset R$ are unknown (dependent on θ). The most logical approach is, once again, resorting to permutation tests.

## 3 Distance Correlation–Based Test for Epistasis

Thus far, the theoretical basis for the usage of distance correlation within certain metric spaces has been set. Upon this, extensions and applications can be formulated. There are a few examples of this in the literature, in contexts such as time series (Davis et al. 2018), topological data analysis (Turner and Spreemann 2020), and discretized stochastic processes (Dehling et al. 2020).

What is about to be presented is the particularization of the theoretical framework to spaces of cardinality 3, to then design a procedure that is adapted to epistasis detection and that, at the same time, solves the inadequacy of the tests by Cai and Liu (2016) to this setting (as shown in Figure 1).

### 3.1 Distance Correlation in Spaces of Cardinality 3

Clearly, in a finite space, the finiteness of moments (of any order) and separability are not an issue. In addition, one can resort to brute force and solve the system of inequations that are derived from simply using the definitions (Klebanov 2005; Lyons 2013), obtaining a direct—albeit cumbersome— proof of the fact that any 3‐point space $\left(X,d_{X}\right)$ is necessarily of strong negative type. Such proof is, in principle, superfluous, as long as one wants to make use of strong theorems, such as Schoenberg's: $\left(X,\sqrt{d_{X}}\right)$ can clearly be embedded into a Hilbert space, isometric to the vertices of a triangle in $R^{2}$ (note that the square root transformation preserves the triangle inequality of the metric). Nevertheless, it is interesting to check that, when the metric structure becomes so simple, abstract arguments (such as the ones that arise in the proof of Schoenberg's theorem) become unnecessary.

Let X:={0,1,2} be the set of the three possible genotypes for each SNP. There is no biological reason to assume that 2∈X copies of the minor allele affect twice as much as one (Bush and Moore 2012), neither when it comes to increasing the susceptibility to a psychiatric disorder nor to decreasing it. As a matter of fact, in some cases, this susceptibility is maximal under heterozygosis (Costas et al. 2011), which is coded by 1∈X.

Therefore, there is no rationale for prioritizing the Euclidean distance

d(0,2)=2d(0,1)=2d(1,2)

over more general (non‐“linear”) metric spaces. And this is why distance correlation turns out to be a sensible way to adapt the ideas of Cai and Liu (2016) to our setting. As previously commented, the marked discreteness of SNP data provides another incentive for transcending the idea of linear correlation.

No specific type of interaction is being looked for—the aim of this article is to simply detect epistasis. For this reason, we will henceforward focus on the *equilateral distance* (also known as the *discrete metric*):

d(0,1)=d(1,2)=d(0,2)=1.

Three further conspicuous distances are to be defined, which are the degenerate ones (in which two of the vertices of the triangle are the same point), that can be especially illustrative in the analysis of real data because they allow a straightforward interpretation of the allelic model that is being studied:

(1) Recessive (distance “0 = 1”): d(0,1)=0;d(0,2)=d(1,2)=1.
(2) Heterozygous (distance “0 = 2”): d(0,2)=0;d(0,1)=d(2,1)=1.
(3) Dominant (distance “1 = 2”): d(1,2)=0;d(1,0)=d(2,0)=1.

Technically, the three previous distances are not metrics, but rather pseudometrics, as they yield d(x,y)=0 for x≠y. However, one can modify the labels on the initial 3‐point space so that it becomes a 2‐point space, in which case the former pseudometric is an actual metric for which all the theoretical results of Jakobsen (2017) immediately follow. More importantly, distance covariance could also be defined for semimetric spaces (i.e., by dropping the triangle inequality), using the definition by Sejdinovic et al. (2013). Therefore, having a premetric space would suffice for a notion of generalized distance covariance to exist.

### 3.2 Construction of a Hypothesis Test

Searching for epistasis consists in looking for significantly different dependence structures between the case and control groups, as previously discussed. To simplify notation, let $Z_{i}$ and $Z_{j}$ be random variables with support $Z\in \left\{X,Y\right\}$, corresponding to two different SNPs, for which a joint sample of size $n\in Z^{+}$ is available:

$$\left(Z_{i,1},Z_{j,1}\right),\text{…},\left(Z_{i,n},Z_{j,n}\right)\;\text{IID}\;\left(Z_{i},Z_{j}\right).$$

The aim is to test the independence of $\left\{Z_{i},Z_{j}\right\}$ or, equivalently,

$$\left\{\begin{array}{c} H_{0ij}:\mathrm{dcov}\left(Z_{i},Z_{j}\right)=0\\ H_{1ij}:\mathrm{dcov}\left(Z_{i},Z_{j}\right)\neq 0\;; \end{array}\right.$$

which extends the large‐scale multiple tests of local covariance and correlation structures (see Supporting Information Appendix C.1) to the use of distance covariance and correlation, instead of their product‐moment counterpart.

In order to approximate the null distribution of the test statistic $\hat{\mathrm{dcov}}\left(Z_{i},Z_{j}\right)$, one can take advantage of the beauty of finite marginal spaces, in which only a few of the coefficients of the quadratic form that gives the asymptotic null distribution of distance covariance will be non‐null. Namely, we present two theorems for such distributions, both for the metric of maximum interest to us and for the Euclidean distance (i.e., for classical distance covariance). Proofs can be found in Supporting Information Appendix B.

Theorem 3.1 Let $\left(X_{1},\text{…},X_{n}\right)$ and $\left(Y_{1},\text{…},Y_{n}\right)$ be IID samples of jointly distributed random variables (X,Y)∈{0,1,2}×{0,1,2}, with $p_{j}:=P\left(X=j\right)$ and $q_{j}:=P\left(Y=j\right)$, for j∈{0,1,2}.

Consider X=Y={0,1,2} equipped with the discrete metric.

Then, whenever X and Y are independent, for n→∞,

$$n\;{\hat{\mathrm{dCov}}}_{\text{discrete}}^{2}\overset{D}{\underset{n\to \infty }{⟶}}\lambda_{1}\;\mu_{1}Z_{11}^{2}+\lambda_{1}\mu_{2}Z_{12}^{2}+\lambda_{2}\mu_{1}Z_{21}^{2}+\lambda_{2}\mu_{2}Z_{22}^{2},$$

where $Z_{11}^{2},\;Z_{12}^{2},\;Z_{21}^{2}$, and $Z_{22}^{2}$ are independently chi‐squared distributed with one degree of freedom. $\lambda_{1}$ and $\lambda_{2}$ are given by

(3) $$\begin{array}{ccc} \lambda_{1,2} & = & \frac{1-\sum p_{j}^{2}}{2}\pm \sqrt{\frac{{\left(1-\sum p_{j}^{2}\right)}^{2}}{4}-3\prod p_{j}}. \end{array}$$

Similarly, $\mu_{1}$ and $\mu_{2}$ are given by

(4) $$\begin{array}{ccc} \mu_{1,2} & = & \frac{1-\sum q_{j}^{2}}{2}\pm \sqrt{\frac{{\left(1-\sum q_{j}^{2}\right)}^{2}}{4}-3\prod q_{j}}. \end{array}$$

 □

Theorem 3.2 Let $\left(X_{1},\text{…},X_{n}\right)$ and $\left(Y_{1},\text{…},Y_{n}\right)$ be IID samples of jointly distributed random variables (X,Y)∈{0,1,2}×{0,1,2}, with $p_{j}:=P\left(X=j\right)$ and $q_{j}:=P\left(Y=j\right)$, for j∈{0,1,2}.

Consider X=Y={0,1,2} equipped with the Euclidean metric.

Then, whenever X and Y are independent, for n→∞,

$$n\;{\hat{\mathrm{dCov}}}_{\text{Euclidean}}^{2}\overset{D}{\underset{n\to \infty }{⟶}}\lambda_{1}\;\mu_{1}Z_{11}^{2}+\lambda_{1}\mu_{2}Z_{12}^{2}+\lambda_{2}\mu_{1}Z_{21}^{2}+\lambda_{2}\mu_{2}Z_{22}^{2},$$

where $Z_{11}^{2},Z_{12}^{2},Z_{21}^{2},and\;Z_{22}^{2}$ are independently chi‐squared distributed with one degree of freedom. $\lambda_{1}$ and $\lambda_{2}$ are given by

$$\begin{array}{ccc} \lambda_{1,2} & = & p_{0}\left(1-p_{0}\right)+p_{2}\left(1-p_{2}\right)\\ & & \pm \sqrt{{\left(p_{0}\left(1-p_{0}\right)+p_{2}\left(1-p_{2}\right)\right)}^{2}-4\prod p_{j}}. \end{array}$$

Similarly, $\mu_{1}$ and $\mu_{2}$ are given by

$$\begin{array}{ccc} \mu_{1,2} & = & q_{0}\left(1-q_{0}\right)+q_{2}\left(1-q_{2}\right)\\ & & \pm \sqrt{{\left(q_{0}\left(1-q_{0}\right)+q_{2}\left(1-q_{2}\right)\right)}^{2}-4\prod q_{j}}. \end{array}$$

 □

We will now show that Theorems 3.1 and 3.2 induce asymptotic tests for testing the null hypothesis that X and Y are independent.

Corollary 3.3 Let $\left(X_{1},\text{…},X_{n}\right)$ and $\left(Y_{1},\text{…},Y_{n}\right)$ be IID samples of jointly distributed random variables (X,Y)∈{0,1,2}×{0,1,2}. For each j∈{0,1,2}, we define

$${\hat{p}}_{j}:=\frac{1}{n}\sum_{i=1}^{n}1_{\left\{X_{i}=j\right\}};\;{\hat{q}}_{j}=\frac{1}{n}\sum_{i=1}^{n}1_{\left\{Y_{i}=j\right\}},$$

and let ${\hat{\lambda }}_{1,2}$ and ${\hat{\mu }}_{1,2}$ be the plug‐in versions of Equations (3) and (4) by ${\hat{p}}_{j}$ and ${\hat{q}}_{j}$, respectively, that is,

$${\hat{\lambda }}_{1,2}=\frac{1-\sum {\hat{p}}_{j}^{2}}{2}\pm \sqrt{\frac{{\left(1-\sum {\hat{p}}_{j}^{2}\right)}^{2}}{4}-3\prod {\hat{p}}_{j}},$$

$${\hat{\mu }}_{1,2}=\frac{1-\sum {\hat{q}}_{j}^{2}}{2}\pm \sqrt{\frac{{\left(1-\sum {\hat{q}}_{j}^{2}\right)}^{2}}{4}-3\prod {\hat{q}}_{j}}.$$

Denoting the joint sample by $S=\{\left(X_{1},Y_{1}\right),\text{…},\left(X_{n},Y_{n}\right)\}$, given a nominal level α∈]0,1[, we define the threshold ${\hat{c}}_{\alpha }\left(S\right)$ as the largest nonnegative number satisfying

$$P(\hat{\lambda_{1}}\;{\hat{\mu }}_{1}Z_{11}^{2}+\hat{\lambda_{1}}{\hat{\mu }}_{2}Z_{12}^{2}+\hat{\lambda_{2}}\hat{\mu_{1}}Z_{21}^{2}+{\hat{\lambda }}_{2}{\hat{\mu }}_{2}Z_{22}^{2}\geq {\hat{c}}_{\alpha }\left(S\right)\;|S)=\alpha ,$$

where we are simplifying the notation by writing $\hat{\lambda_{1}}\equiv \hat{\lambda_{1}}\left(S\right)$ and so forth. Then, if X and Y are independent, it holds that

$$P(n\;{\hat{\mathrm{dCov}}}_{\text{discrete}}^{2}\left(S\right)\geq {\hat{c}}_{\alpha }\left(S\right))⟶\alpha ,$$

as n→∞. Hence the function

$$\varphi :S\mapsto P\left\{{\hat{\mathrm{dCov}}}_{\text{discrete}}^{2}\left(S\right)\geq {\hat{c}}_{\alpha }\left(S\right)\right\}$$

is an asymptotic test of size α for testing the nonparametric null hypothesis

$$H_{0}:X\;\text{and}\;Y\;\text{are independent}$$

against the alternative

$$H_{1}:X\;\text{and}\;Y\;\text{are not independent.}$$

□

The only remaining task is to calculate the quantiles ${\hat{c}}_{\alpha }$, for which efficient algorithms are available (Duchesne and Lafaye de Micheaux 2010).

An analogous result to Corollary 3.3 holds for the test based on $n\;{\hat{\mathrm{dCov}}}_{\text{Euclidean}}^{2}$, which would also work by plugging in the allele frequencies into the expression of the coefficients of the Gaussian quadratic form.

It is crucial to note that we do not want to directly test for the equality of distance correlations, as Cai and Liu (2016) did when looking for differential gene co‐expression, following the rationale by De la Fuente (2010) and others. In our search for epistasis, however, we are just interested in finding SNP pairs that are dependent for the cases and independent for the controls. When, for some SNP pair, independence is rejected for healthy individuals and not for patients, it will be attributed to a spurious interaction resulting from *population substructure* (Brandes et al. 2022), that is, from the effect of unmeasured (and often unmeasurable) covariates.

## 4 Simulations

In order to validate our testing procedure, we have designed some population models in which the intensity of dependence can be adjusted by tuning a parameter. We first introduce those models, to then use them to compare the performance of our method with that of BOOST (Wan et al. 2010), one of the most popular epistasis detectors within the genomics community. For distance covariance, we will consider the discrete metric in every scenario because it reflects our agnosticism on the underlying genetic model.

### 4.1 Design of Population Models for the Validation of Our Methodology

The theoretical models that are about to be defined refer to the interaction between an arbitrary pair $\left\{Z_{i},Z_{j}\right\}$, where Z is either X or Y, depending on the case. When it came to setting the marginal frequencies, instead of allowing for two degrees of freedom on each marginal, a further restriction was introduced (apart from the sum being one): allele and genotype frequencies were constrained to be in Hardy–Weinberg equilibrium (Hardy 1908), since all the SNPs in the schizophrenia database verify it (it is one of the quality controls that are used). Hence, there is a single free parameter, which is the minor allele frequency, that is sampled from a uniform distribution on [0.05,0.2]. The lower limit mimics standard GWAS quality control filters (in settings with moderate sample size), and the upper one was set so that the resulting true interactions are not the easiest to detect.

There are a few options in the literature for simulating epistasis between SNPs. Some models (like the ones by Marchini et al. 2005) are overly simplistic, for example, by not allowing to adjust the interaction intensity in order to assess the robustness against different alternatives. Some recent approaches (like the ones studied by Russ et al. 2022) make interpretability more difficult, in the sense that we are very interested in quantifying the intensity of interaction (i.e., deviation from the null hypothesis) when assessing the power of our test. In order to overcome such shortcomings, we introduce our own models for SNP–SNP interaction.

The most straightforward model is one in which the probability of each genotype is the product of the marginals (there is independence). For dependence, two kinds of models will be defined. On the one hand, model qexp conveys a dependence structure that becomes more intense as parameter e∈[1,+∞[ increases, in the way that Table 1 describes. On the other hand, model qmult has g∈[0,1] as its free parameter (Table 2). Again, the closer the parameter is to 1, the less notorious the association becomes.

**TABLE 1:** Contingency table for model qexp.

$Z_{i}$ \$\;Z_{j}$	0	1	2	
0	$pr+q^{e}s-qs$	$ps-q^{e}s+qs$	p(1−r−s)	p
1	$qr-q^{e}s+qs$	$q^{e}s$	q(1−r−s)	q
2	(1−p−q)r	(1−p−q)s	(1−p−q)(1−r−s)	1−p−q
	r	s	1−r−s	1

**TABLE 2:** Contingency table for model qmult.

$Z_{i}$ \$\;Z_{j}$	0	1	2	
0	pr−(1−g)qs	ps+(1−g)qs	p(1−r−s)	p
1	qr+(1−g)qs	gqs	q(1−r−s)	q
2	(1−p−q)r	(1−p−q)s	(1−p−q)(1−r−s)	1−p−q
	r	s	1−r−s	1

### 4.2 Results of the Simulation Study

Each simulation consisted in the study of one of the models for a SNP pair. This is an acceptable simplification because the current setting is a problem of multiple testing and not a single high‐dimensional test (see Supporting Information Appendix C.1). This means that it is not necessary to build and replicate a whole n×L matrix, and that it suffices to perform replications at the SNP‐pair level.

**FIGURE 2:**
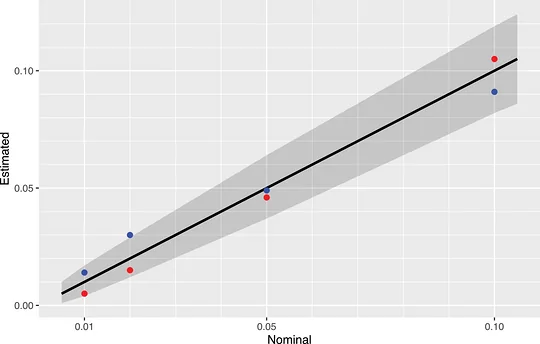
Empirical power under the null hypothesis ($\hat{\alpha }$) versus nominal significance level (α), under model indep in cases and indep in controls. Blue dots correspond to *dcov*; the red ones were generated with BOOST. The gray shadow is a 95 % confidence band for $\hat{\alpha }$ given α.

**FIGURE 3:**
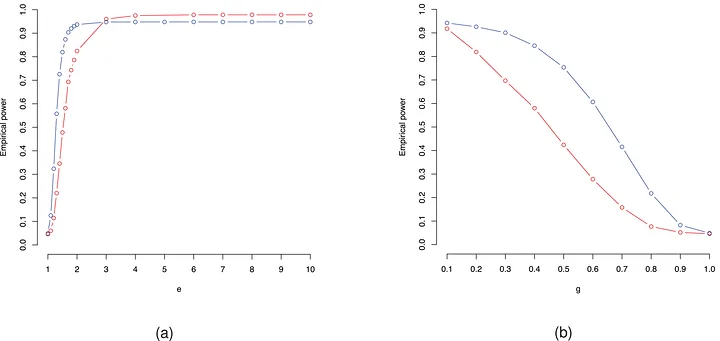
Empirical power when the SNP pair distributions for cases/controls are (a) qexp with parameter $e\in Z^{+}$ and indep and (b) qmult with parameter g∈[0,1] and indep. Color blue represents our distance covariance test, whereas red corresponds to BOOST.

We now briefly show some simple, illustrative examples of the performance of our testing procedure. First, Figure 2 shows the calibration of significance for some usual nominal levels for the only scenario under the null hypothesis we expect to come across in practice, that is, independence in both cases and controls. On the other hand, empirical power is represented in Figure 3. In all cases, R=1000 replicates were carried out. The sample size in each replicate, for each of the control and case group, is 500 plus a perturbation that is uniformly drawn from the integer interval [−10,10]∩Z, in order to demonstrate the applicability of our method in scenarios where $n_{1}\neq n_{2}$.

In each plot, we also display the results we obtained with one of the most popular tools within the genomics community for the kind of epistasis we are studying—it is called *BOOST* (Wan et al. 2010) and is easily accessible from the widely used genetics software package PLINK (Purcell and Chang 2023). As indicated in Supporting Information Appendix A, there is an extremely large number of options in the literature to perform this task and, therefore, it is not feasible to compare our technique with a representative fraction of them.

On the basis of Figure 2, it can be concluded that the calibration of significance is acceptable or even good for the most usual levels of nominal α. In addition, the plots in Figure 3 show that the power is very satisfactory and that, as expected, it increases as one gets further away from the null hypothesis. In the scenarios we studied, we have either comparable or more power than BOOST.

## 5 Application to a Case‐Control Study of Schizophrenia

The genomic database that we study in this article is described in detail in Supporting Information Appendix F for the sake of reproducibility. It contains observations of 6,371,078 SNPs across the entire genome, from a case‐control study of schizophrenia in Galicia (Rodríguez‐López et al. 2020), with $n_{1}=585$ cases and $n_{2}=573$ controls. For a better understanding of the nature of the dataset and the quality controls (Ziegler et al. 2008) and downstream analysis it underwent, we refer the reader to Supporting Information Appendices F and G. Supporting Information Appendix G also contains further details on reproducibility.

In Section 5.1, we illustrate the testing procedure for genetic interaction, for which we set the theoretical basis in Section 3 and whose numerical performance we examined in Section 4. Then, in Sections 5.2 and 5.3, we present two experimental setups we carried out to better understand the performance of our methodology in biostatistical practice. In them, we use our DNA data as the starting point for interpretations at “higher” levels of the biomolecular hierarchy (proteins and RNA), based on missense SNPs and genetically regulated gene expression, respectively. As with the simulations, we restrict ourselves to the discrete metric in order to be agnostic regarding the underlying genetic model. In the latter two experiments, we apply our methodology to SNP pairs across the human genome, to then interpret the results by performing several tests comparing proportions and ranks in cases versus controls. The key rationale is that the set of putative interactions detected with our testing procedure (i.e., the SNP pairs for which independence is rejected in cases and not in controls) will include both pairs in “true” epistasis and noise, whereas the SNP pairs where independence is rejected in controls and not in cases are just spurious interactions (due to population substructure).

### 5.1 Illustration of the Distance Correlation–Based Test for Genetic Interaction

As an example of how our testing procedure would work out when testing for epistasis between two SNPs, let us consider SNP rs6972158 (in chromosome 7) and SNP rs61749697 (chromosome 20). These are missense SNPs (i.e., the variation of that nucleotide changes the amino acid sequence of the protein encoded by the gene) in genes *NPSR1* and *GNAS*, respectively. *NPSR1* codes for a G‐protein‐coupled receptor for neuropeptide S. *GNAS* codes for a protein that binds to many different G‐protein‐coupled receptors, being essential for the transduction of cellular signals. Using our schizophrenia dataset and applying the distance covariance test, we get a p‐value of 0.041 in cases and of 0.966 in controls. This means that, for a nominal α of 0.05, we reject independence in cases and not in controls. Then this pair would be in putative epistasis.

In order to check if this makes sense biologically, we conducted a search in the STRING database version 12.0 (Szklarczyk et al. 2023). According to the interaction prediction pipeline of STRING, based on several sources of evidence, the combined confidence score of the functional interaction between these two proteins is 0.619, regarded as a medium level. Confidence scores are scaled between zero and one and correspond to the estimated likelihood of the association under consideration being true, given the underlying evidence. Therefore, the putative interaction between these two SNPs detected by our method is biologically plausible.

### 5.2 Experiment I: Functional Enrichment

Taking into account the goal of this first experiment, for the reasons stated in Supporting Information Appendix G, it is sensible to restrict ourselves to a certain subset of the initial database, comprising L=8030 missense SNPs.

First, we apply our test procedure separately to cases and controls (as previously discussed), with a Benjamini and Hochberg (1995) nominal threshold for the false discovery rate (FDR) of 0.05. We only consider SNP pairs consisting of two variants that lie on different chromosomes or that are more than 1 Mb apart (i.e., not physically close). This prevents evident cases of spurious findings due to linkage disequilibrium (Wan et al. 2010).

We thus obtain 113 out of L2 SNP pairs that show association in cases and not in controls (which we consider putative interactions), versus 95 in controls and not in cases (which just reflect population substructure). The difference (in proportions) is not significant; with a p‐value of 0.12, which is greater than any conventionally used nominal value of α. These 113 and 95 pairs correspond, respectively, to 222 and 189 unique SNPs, a proportion difference with p of 0.055. Those SNPs lie on 220 and 191 different genes. Removing the 13 that are common among both lists, we get 207 and 178 genes (p of 0.07).

We hypothesize that there will be an overrepresentation of genes involved in synapse, which is a brain function known to be closely related to schizophrenia (Koopmans et al. 2019), in our group of putative interactions with respect to the spurious ones. Intersecting, we see that 13 of the 207 and 9 out of 178 genes are known to be related to synapse. The proportion difference has a p‐value of 0.26, so our results for this part are negative. We can show no (strong) evidence that we are detecting any signal related to synapse.

However, this is not to say that our method cannot offer interesting insight into these data. The current knowledge on complex disease genetics indicates that regulatory regions play a crucial role (Sullivan and Geschwind 2019), so one should focus on genetically regulated gene expression, rather than on missense SNPs. This motivates Experiment II.

### 5.3 Experiment II: Gene Expression

With this second data example, we want to show that our results make sense at the level of genetically regulated gene expression (i.e., mRNA). For this task, as explained in the Supporting Information, only some variables in our schizophrenia database can be used, comprising some L=6456 SNPs that regulate gene expression in the brain, but not in any other tissue of the human body, according to data from the GTEx Consortium (2023).

We now apply our procedure as in Experiment I, seeing that there are significantly more pairs in putative interaction than in spurious one: 1272 versus 1137 (with *p* of 0.032), after applying the physical distance threshold of 1 Mb. These pairs represent 1539 and 1439 unique SNPs, respectively, again a significant difference (p‐value ≈0.019, which drops to 0.0024 by removing SNPs in both sets).

We finally order the p‐values we obtained for each of the L2 tests we performed on cases, and do the same for controls. We then take the absolute difference of both ranks for each SNP pair. We hypothesize that these absolute rank differences will tend to be greater on the true positive list than on the false positives. We perform a Wilcoxon–Mann–Whitney U test, and we find that we can confirm that it is the case, with a p‐value of less than $2.2\times 10^{-16}$.

All in all, the results of Experiment II indicate that we are detecting some genuine signal at the level of genetically regulated gene expression. This would be very unlikely if our method did not function correctly.

## 6 Discussion and Conclusion

Distance correlation has been shown to characterize independence in certain metric spaces, establishing how this theory is valid for the case of 3‐point marginal spaces. With this approach, a hypothesis test based on the general characterization of independence that distance correlation offers has been designed, extending the idea of LCTs (Cai and Liu 2016) to ternary data.

We derive the explicit asymptotic null distribution of the distance‐covariance statistics that arise. To our knowledge, the usage of distance correlation in discrete spaces (in genomics or elsewhere)—and, in particular, its application to the search for SNP–SNP interactions—has no precedents in the literature. Moreover, no previously published research has attempted to perform large‐scale multiple testing with any of the techniques derived from energy statistics (Székely and Rizzo 2017). However, what does exist in the literature is the usage of distance correlation for finding the association between genetic data (as observations of continuous random variables in Euclidean spaces) and a phenotype (Hua and Ghosh 2015), which is another interesting problem, but completely different both regarding biological and mathematical factors.

Simulations show that the calibration of significance is adequate and that power is considerably high against various alternatives. We also show that we generally outperform Wan et al. (2010)—one of the most popular epistasis detectors—in the scenarios we have studied.

The schizophrenia database has been analyzed with our methodology, obtaining biologically sound results at the level of genetically regulated gene expression. Some recent studies show evidence of epistasis between regulatory regions of the human genome (Lin et al. 2022; Patel et al. 2022), which supports our findings.

In order to frame our results, we would like to emphasize that all popular epistasis detectors find a large number of false positives and do not have really high power (Russ et al. 2022). Therefore, the main limitation of our method (as it is of any other for this task) is that it is very difficult to make any solid discoveries when working with real data. Epistasis detection is an extremely challenging biostatistical problem, in which there is still much progress to be made, given its key role in human complex genetics (van Steen and Moore 2019).

Future work may include investigating further distances, like the pseudometrics at the end of Section 3.1. Another interesting point would be to adapt our techniques to the search for interactions between the mitochondrial (haploid) and nuclear (diploid) genome of an individual, that is, to the study of independence between binary and ternary variables. More generally, a promising line of future research could be the extension of the philosophy of our current paper to the categorical testing for two variables with an arbitrary number of categories, as in Castro‐Prado et al. (2026). Finally, one could even aim at testing for independence between SNP data and a phenotype of interest (regardless of it being a continuous, binary, or survival‐type outcome), which would amount to testing for marginal effects of individual variants, which is a main goal of GWASs (Abdellaoui et al. 2023).

## Conflicts of Interest

The authors declare no conflicts of interest.

## Open Research Badges

This article has earned an Open Data badge for making publicly available the digitally‐shareable data necessary to reproduce the reported results. The data is available in the Supporting Information section.

This article has earned an open data badge “**Reproducible Research**” for making publicly available the code necessary to reproduce the reported results. The results reported in this article could fully be reproduced.

## Supporting information


**Supporting File 1:** bimj70150‐sup‐0001‐SuppMat.pdf.

**Supporting File 2:** bimj70150‐sup‐0002‐Code.zip.

## Data Availability

The data that support the findings of this study are openly available in Zenodo at http://doi.org/10.5281/zenodo.20744952, as well as in the supplementary material of this article.
