# Supplementary material for: Testing for Genetic Interactions in Complex Disease With Distance Correlation
Source: Biom J. 2026 Sep 7;68(5):e70150. doi: 10.1002/bimj.70150 (PMC13548348; doi:10.1002/bimj.70150)
Supplement: Supplementary file 1 [file BIMJ-68-e70150-s002.pdf]

Biometrical Journal **68** (2026), *e70150*. DOI: 10.1002/bimj.70150

Supplement to: Testing for genetic interactions in complex disease with distance correlation

Fernando Castro-Prado [*,1,2,3], **Javier Costas** [2], **Dominic Edelmann** [3], **Wenceslao González-Manteiga** [1], and **David R. Penas** [1,4]

[1] Department of Statistics, Faculty of Mathematics, University of Santiago de Compostela, Galicia, Spain.
[2] Psychiatric Genetics Laboratory, Santiago Health Research Institute (IDIS), University Hospital, Santiago de Compostela, Galicia, Spain.
[3] Biostatistics Department, German Cancer Research Center (DKFZ), Heidelberg, Baden-Württemberg, Germany.
[4] Computational Biology Laboratory, Spanish National Research Council (MBG-CSIC), Pontevedra, Galicia, Spain.

This supplement contains additional material to the main body of our article.

*Corresponding author: e-mail: f.castro.prado@usc.es

© 2026 WILEY-VCH Verlag GmbH & Co. KGaA, Weinheim

A Statistical approaches to epistasis detection

The recent development of the "-omic" disciplines has been parallel to the creation of bioinformatic tools to process the vast amount of data that these experimental sciences produce. The diversity of the available "-omic" software is so large that it has even been necessary to develop meta-tools to index the existing techniques. For example, the directory of one of them (Henry *et al.*, 2014) contains more than 20 000, 900 of which are designed for GWAS data analyses, which in turn contain a subset of 100 that are suitable for epistasis detection.

The existence of such a large number of proposed solutions for such a specific task owes to the surprisingly wide variety of statistical methods that are valid for it—e.g. linear models (standard and generalized), logistic regression, tests on Pearson's correlations, permutation tests, Bayesian inference, random forests, Markov chains, co-information indices, graph theory, and maximal entropy probability models.

Another cause of that diversity of alternatives is the fact that some of the available techniques only focus on a specific subproblem (pairwise gene-gene interactions versus higher orders, binary versus continuous response variable, pedigrees, stratified populations and so forth) and that many different computing strategies can be used to yield results in reasonable amounts of time (for instance, biology-based initial filters, code parallelization, graphical processing units, Boolean operations, machine learning approaches, and ant colony optimization algorithms).

Table S1 Some remarkable epistasis detection tools for GWAS data analysis

Tool	Statistical techniques	Computational tricks	Reference
2S-LRM	Logistic regression	Pre-filtering	Pecanka *et al.* (2017)
AntEpiSeeker	χ^2 tests	Ant colony optimization	Wang *et al.* (2010)
BEAM	Bayesian MCMC	None	Zhang and Liu (2007)
BOOST	Logistic regression	Boolean operations, parallelization	Wan *et al.* (2010a)
BiForce	Linear regression	Boolean operations, parallelization	Gyenesei *et al.* (2012)
CES	Evolutionary algorithms	Artificial intelligence	Moore and Hill (2015)
CINOEDV	Information theory	Swarm intelligence on hypergraphs	Shang *et al.* (2016)
EpiGPU	Linear regression	GPU architectures	Hemani *et al.* (2011)
EpiACO	Information theory	Ant colony optimization	Sun *et al.* (2017)
EpiBlaster	Pearson's correlations	GPU architectures	Kam-Thong *et al.* (2011)
Fiúncho	Information theory	Parallelization	Ponte *et al.* (2022)
GLIDE	Linear regression	GPU architectures	Kam-Thong *et al.* (2012)
GWIS	ROC curve analysis	GPU architectures	Goudey *et al.* (2013)
IndOR	Logistic regression	Pre-filtering	Emily (2012)
MDR	Combinatorics, resampling	Pre-filtering	Ritchie *et al.* (2001)
Random Jungle	Random forests	Parallelization	Schwarz *et al.* (2010)
SNPruler	Information theory	Branch and bound algorithms	Wan *et al.* (2010b)
Stage-wise LRT	GLMs, closed testing	Hierarchical testing	Frånberg *et al.* (2015)
Wtest	Logistic regression	None	Sun *et al.* (2019)

Table S1 summarizes some of the existing methods, including the ones reviewed by Gusareva and van Steen (2014), Niel *et al.* (2015) and Russ *et al.* (2022) and some others that we consider representative. Some are very widely used, like BOOST, due to its being implemented in the popular genetics toolset PLINK (Purcell et al., 2023); whereas other methods in the table have not been used much in practice.

© 2026 WILEY-VCH Verlag GmbH & Co. KGaA, Weinheim

B Derivation of the asymptotic null distribution of the test statistic

We first state, as Lemma B.1, a result by Edelmann and Goeman (2022) that is instrumental in the proof of Theorem 3.1, which is the central theoretical result of our article. When it comes to proving Theorem 3.2 (which we do not use in simulations nor in real data analyses), we make use of work by Huang and Huo (2022). We refer the reader to that article —instead of reproducing its content here—, in order to stay on topic. Finally, we provide the proof of Corollary 3.3., which is the key result for the application of our methodology.

B.1 A lemma for the discrete metric

Lemma B.1 (Edelmann and Goeman [2022], Theorem 7) *Let X and Y be random variables with supports $\{1,2,\dots,I\}$ and $\{1,2,\dots,J\}$, respectively; where $I,J\in\mathbb{Z}^+$.*

We now construct the entries of matrix $\mathbf{L}^X=(L^X_{rs})_{r,s}\in\mathbb{R}^{I\times I}$ as follows:

$$L^X_{rs}=p_s\left(\delta_{rs}-p_r-p_s+\sum_{i=1}^{I}p_i^2\right),$$

where $\delta_{\cdot\cdot}$ is the Kronecker delta and $p_i:=\mathrm{P}(X=i)$ is the probability mass of X in $i\in\{1,\dots,I\}$. Furthermore, let us denote by

$$\lambda_1^X,\dots,\lambda_{I-1}^X$$

the nonzero eigenvalues of $\mathbf{L}^X$. If $\{\lambda_j^Y\}_{j=1}^{J-1}$ are defined analogously, then the following limit in distribution holds under independence of X and Y, as $n\to\infty$:

$$n\,\widehat{\mathrm{dCov}}^{\,2}_{\textit{discrete}}(X,Y)\xrightarrow{\mathcal{D}}\sum_{i=1}^{I-1}\sum_{j=1}^{J-1}\lambda_i^X\lambda_j^Y Z_{ij}^2;$$

with $\{Z_{ij}\}_{i,j}$ being IID standard Gaussian.

B.2 Proof of Theorem 3.1 (discrete metric)

Applying Lemma B.1, one gets that, as $n\to\infty$,

$$n\,\widehat{\mathrm{dCov}}^{\,2}_{\text{discrete}}(X,Y)\xrightarrow{\mathcal{D}}\lambda_1\,\mu_1 Z_{11}^2+\lambda_1\mu_2 Z_{12}^2+\lambda_2\mu_1 Z_{21}^2+\lambda_2\mu_2 Z_{22}^2,$$

where $\{Z_{ij}^2\}_{i,j}$ are IID χ_1^2; whereas $\{\lambda_j\}_{j=1}^2$ and $\{\mu_j\}_{j=1}^2$ are the nonzero eigenvalues of certain matrices. Namely, λ_1 and λ_2 are the non-null eigenvalues of the following matrix:

$$\mathbf{A}=\begin{pmatrix}(1-2p_0+\sum p_j^2)\,p_0 & (-p_0-p_1+\sum p_j^2)\,p_1 & (-p_0-p_2+\sum p_j^2)\,p_2\\ (-p_0-p_1+\sum p_j^2)p_0 & (1-2p_1+\sum p_j^2)\,p_1 & (-p_1-p_2+\sum p_j^2)\,p_2\\ (-p_0-p_2+\sum p_j^2)p_0 & (-p_1-p_2+\sum p_j^2)\,p_1 & (1-2\,p_2+\sum p_j^2)\,p_2\end{pmatrix}.$$

By multiplying each of the rows of $\mathbf{A}$ by p_0, p_1 and p_2 respectively, and adding up the rows, one easily sees that this matrix is singular. Using the relation $p_0+p_1+p_2=1$, we can write the characteristic polynomial of $\mathbf{A}$ as:

$$P(\lambda)=\lambda\left(\lambda^2-\left(1-\sum_{j=1}^{3}p_j^2\right)\lambda+3\prod_{j=1}^{3}p_j\right),$$

where $P(\lambda)$ is defined using the sign convention that makes it monic: $\det(\lambda\,\mathbf{I}_3-\mathbf{A})$.

Calculating the roots of $P(\lambda)$ yields λ_1 and λ_2. The derivation of μ_1 and μ_2 follows the same strategy.

© 2026 WILEY-VCH Verlag GmbH & Co. KGaA, Weinheim

B.3 Proof of Theorem 3.2 (Euclidean distance)

Throughout the proof, we will use the notation:

$$M := 2p_0p_1 + 2p_1p_2 + 4p_0p_2 = 2p_0(1-p_0) + 2p_2(1-p_2).$$

Applying Theorem 4.12 and Lemma 4.14 in Huang and Huo (2022), we obtain that, when $n \to \infty$,

$$n\,\widehat{\mathrm{dCov}}^2_{\text{Euclidean}}(X,Y) \xrightarrow{\mathcal{D}} \sum_{i,j=1}^{3} \lambda_i \mu_j Z_{ij}^2,$$

as long as X and Y are independent. In the above expression, $\{Z_{ij}^2\}_{i,j}$ are IID χ_1^2 and $\{\lambda_j\}_{j=1}^3$ are the eigenvalues (all of them nonnegative real numbers) of the following matrix:

$$\begin{pmatrix} (2p_1+4p_2-M)\,p_0 & (-1+p_1+3p_2-p_0+M)\,p_1 & M\,p_2 \\ (-1+p_1+3p_2-p_0+M)p_0 & (2p_2+2p_0-M)\,p_1 & (-1+3p_0+p_1+p_2-M)\,p_2 \\ Mp_0 & (-1+3p_0+p_1+p_2-M)\,p_1 & (2p_1+4p_0-M)\,p_2 \end{pmatrix}.$$

On the other hand, $\{\mu_j\}_{j=1}^3$ are defined analogously. Computing the characteristic polynomial and proceeding as we did for Theorem 3.1 completes the current proof.

B.4 Proof of Corollary 3.3

As a consequence of (Bernoulli's) strong law of large numbers and the continuous mapping theorem, for each $k \in \{1,2\}$, we have:

$$\widehat{\lambda_k} \xrightarrow{a.s.} \lambda_k, \qquad \widehat{\mu_k} \xrightarrow{a.s.} \mu_k.$$

as $n \to \infty$. Hence, it follows that

$$\widehat{\lambda_1}\,\widehat{\mu}_1 Z_{11}^2 + \widehat{\lambda_1}\mu_2 Z_{12}^2 + \widehat{\lambda_2}\widehat{\mu_1} Z_{21}^2 + \widehat{\lambda}_2\widehat{\mu}_2 Z_{22}^2 \xrightarrow{a.s.} \lambda_1\,\mu_1 Z_{11}^2 + \lambda_1\mu_2 Z_{12}^2 + \lambda_2\mu_1 Z_{21}^2 + \lambda_2\mu_2 Z_{22}^2,$$

as $n \to \infty$. Therefore, for almost all $\mathcal{S}$, when $n \to \infty$,

$$\widehat{c}_\alpha(\mathcal{S}) \longrightarrow c_\alpha;$$

with c_α being the $(1-\alpha)-$quantile of the limit distribution. Now, by Slutsky's theorem, under H_0,

$$\frac{n\,\widehat{\mathrm{dCov}}^2_{\text{discrete}}}{\widehat{c}_\alpha} \xrightarrow[n\to\infty]{\mathcal{D}} \frac{\lambda_1\,\mu_1 Z_{11}^2 + \lambda_1\mu_2 Z_{12}^2 + \lambda_2\mu_1 Z_{21}^2 + \lambda_2\mu_2 Z_{22}^2}{c_\alpha}.$$

This allows us to conclude that, whenever X and Y are independent, for $n \to \infty$,

$$P\left(\frac{n\,\widehat{\mathrm{dCov}}^2_{\text{discrete}}}{\widehat{c}_\alpha} \geq 1\right) \longrightarrow P\left(\frac{\lambda_1\,\mu_1 Z_{11}^2 + \lambda_1\mu_2 Z_{12}^2 + \lambda_2\mu_1 Z_{21}^2 + \lambda_2\mu_2 Z_{22}^2}{c_\alpha} \geq 1\right) = \alpha.$$

□

© 2026 WILEY-VCH Verlag GmbH & Co. KGaA, Weinheim

C Large-scale correlation tests (LCTs)

In this section of the supplement, we want to provide a short introduction to the main two philosophies of hypothesis testing for high-dimensional covariance, correlation and precision structures (Section C.1). This serves as an introduction to the LCTs by Cai and Liu (2016), a procedure that features prominently in the main article because it is a testing approach similar to ours, but using Pearson's (product-moment) correlation instead of distance correlation. The LCTs are then explained in detail in Sections C.2 through C.4.

Let us first introduce some notation. First, given $L \in \mathbb{Z}^+$ positions of the genome, let $\mathbf{X} = (X_j)_{j=1}^L$ and $\mathbf{Y} = (Y_j)_{j=1}^L$ be the corresponding random vectors containing the genetic information for case and control individuals, respectively. In the setting studied in the main body of our article, each component has support in $\{0, 1, 2\}$, but for the methods presented in this appendix, based on product-moment covariances, let $\mathbf{X}, \mathbf{Y} \in \mathbb{R}^L$.

We denote the correlation matrices for cases and controls as follows.:

$$\mathbf{R}_1 = (\rho_{ij1})_{i,j} \in \mathbb{R}^{L\times L} \quad \text{and} \quad \mathbf{R}_2 = (\rho_{ij2})_{i,j} \in \mathbb{R}^{L\times L},$$

We observe samples $\{\mathbf{X}_k\}_{k=1}^{n_1}$ IID $\mathbf{X}$ and $\{\mathbf{Y}_k\}_{k=1}^{n_2}$ IID $\mathbf{Y}$, which are assumed to be independent of each other. As we are in a high-dimensional setting, we assume L to be large, both in absolute terms and when compared with n_1 and n_2.

C.1 Testing for high-dimensional covariance, correlation and precision structures

As discussed by Cai (2017), there are two main approaches to testing hypotheses on a high-dimensional covariance, correlation or precision matrix. The first one is the *global* testing of a single hypothesis for a very large matrix, originating from random vectors of very high dimension, which introduces evident complications. An example of this would be to test for

$$H_0 : \mathbf{R}_1 - \mathbf{R}_2 = \mathbf{0},$$

which is of clear interest when one wants to test for differential co-expression. Rejecting such a hypothesis, however, provides no information on which pairs of components (in our setting, SNPs) have differential correlation in the two groups (in our setting, cases and controls).

The other one is the multiple testing of *local* covariance, correlation and network structures. This involves testing hypotheses on pairs of the components of the high-dimensional random vector(s) under consideration, a large number of times (in our setting, $(L^2 - L)/2$ times). This comes with statistical and computational challenges that are not present when testing only one of those $(L^2 - L)/2$ hypotheses separately. The example that is most interesting to us is the test

$$\begin{cases} H_{0ij} : \rho_{ij1} = \rho_{ij2} \\ H_{1ij} : \rho_{ij1} \neq \rho_{ij2} \end{cases}$$

for each pair $(i, j) \in ([1, L] \cap \mathbb{Z})^2$ so that $i < j$. We will now focus on the LCTs by Cai and Liu (2016), which are a robust procedure for testing these hypotheses. The next section motivates the need for transcending conventional correlation testing for this problem.

C.2 LCT: classical approach

A scarcely innovative approach would be to stabilize the variance of the sample correlation coefficients via Fisher's Z transformation (*atanh*). One could think of combining this strategy with a procedure that controls the false discovery rate (FDR), such as the ones by Benjamini and Hochberg (1995) or Benjamini and Yekutieli (2001), thus establishing the desired large-scale correlation test (LCT). The main drawback

© 2026 WILEY-VCH Verlag GmbH & Co. KGaA, Weinheim

of this idea is that, when normality is not ensured, the behavior of the test statistic differs from the well-known asymptotic distribution of the Gaussian case. Simulation studies (Cai and Liu, 2016) show that this method performs very poorly (both with Benjamini–Hochberg and Benjamini–Yekutieli), especially when compared with the LCTs that we introduce next.

C.3 LCT with normal approximation (LCT-N)

The first test that Cai and Liu (2016) devised, the LCT-N, is based on the test statistic

$$T_{ij} := \frac{\hat{\rho}_{ij1} - \hat{\rho}_{ij2}}{\sqrt{\frac{\hat{\kappa}_1}{3n_1}\left(1-\tilde{\rho}_{ij}^2\right)^2 + \frac{\hat{\kappa}_2}{3n_2}\left(1-\tilde{\rho}_{ij}^2\right)^2}},$$

where $\hat{\kappa}_1$ and $\hat{\kappa}_2$ are the respective sample kurtoses of $\mathbf{X}$ and $\mathbf{Y}$, and $\tilde{\rho}_{ijl}$ is a thresholded version of $\hat{\rho}_{ijl}$, for $l \in \{1,2\}$; with $\tilde{\rho}_{ij}^2 := \max\{\tilde{\rho}_{ij1}^2, \tilde{\rho}_{ij2}^2\}$.

H_{0ij} will be rejected when $|T_{ij}|$ is greater than a certain threshold $\hat{t}_\alpha \in \mathbb{R}^+$, which depends on the nominal value $\alpha \in]0,1[$ under which one wants to maintain the FDR. The formula for computing $\hat{t}_\alpha$ works under the assumption that the initial distributions are Gaussian, or are not very far away from being so (elliptical contours). Consequently, the LCT-N should not be used in other contexts.

C.4 LCT with bootstrap (LCT-B)

If the distributions of $\mathbf{X}$ and $\mathbf{Y}$ are totally unknown, it is reasonable to use resampling techniques in order to approximate the tail of the distribution of T_{ij}, which determines $\hat{t}_\alpha$. The bootstrap scheme that Cai and Liu (2016) built to this purpose is consistent and leads to a threshold $\hat{t}_\alpha^*$, which defines the LCT-B. This test is supported by strong theoretical results, which were proven in the original 2016 article.

D A resampling approach to the testing procedure

We initially attempted to approximate the p-values of our test by using a permutation-based approach, which is the gold standard in the distance covariance literature (Székely and Rizzo, 2017). We briefly discuss this brute-force approach, as a *negative result* that can be of utility to other scientists. Although theoretically sound, this strategy leads to such high computation times that it is not suitable for most applications. This is particularly true for genomics, which is so data-intensive that not even the high-performance computing (HPC) approach described in the next section makes resampling feasible. Instead, we recommend resorting to asymptotic results, like the ones presented in our article. Nonetheless, in other scenarios (of lower dimensionality, or in which the theoretical derivation of the asymptotic null distribution is not possible), the following approach might be of interest.

In order to approximate the null distribution of the test statistic $\widehat{\mathrm{dcov}}(Z_i, Z_j)$, it is possible to devise a resampling scheme according to the information that is available under the null hypothesis, which in this case is the independence of Z_i and Z_j. As a result, the reasonable thing to do is not to resample from $\{(Z_{i,k}, Z_{j,k})\}_k$, but to do it separately from $\mathcal{Z}_i := \{Z_{i,k}\}_k$ and $\mathcal{Z}_j := \{Z_{j,k}\}_k$ (permutation tests). Thus, it suffices to compute $B \in \mathbb{Z}^+$ statistics of the form

$$\widehat{\mathrm{dcov}}(\mathcal{Z}_i^{*(b)}, \mathcal{Z}_j^{*(b)})$$

to obtain a Monte–Carlo approximation of the sampling distribution of the empirical distance covariance under H_{0ij}. For a faster algorithm, it suffices to permute only one of the marginals:

$$\widehat{\mathrm{dcov}}(\mathcal{Z}_i^{*(b)}, \mathcal{Z}_j).$$

© 2026 WILEY-VCH Verlag GmbH & Co. KGaA, Weinheim

The usage of permutation tests in this context of metric spaces was inspired by the excellent performance of the same scheme in Euclidean spaces (Székely *et al.*, 2007; Székely and Rizzo, 2017; Edelmann *et al.*, 2022). It has the drawback that there is not the same kind of fully-fledged formal justification of consistency as the one for the naive bootstrap (see Arcones and Giné [1992] and page 100 of Jakobsen [2017]), which should not be a source of concern in practice, like in the Euclidean case. Some preliminary experimental checks (data not shown) point to a better performance of permutation tests versus the naive bootstrap approach in the ternary variable setting, but further studies would be needed to verify it.

It should also be clarified that authors such as Cai and Liu (2016) and Székely *et al.* (2007) argue that the number of resamples B is relatively unimportant for their methods to work, as long as it is not extremely small. With this in mind, and also taking into account that the running time is $O(B)$, we decided to use a moderate value for B in the present article, namely the one proposed by Székely *et al.* (2007) as a function of sample size n:

$$B(n) = 200 + \lfloor 5000/n \rfloor,$$

where $\lfloor \cdot \rfloor : \mathbb{R} \to \mathbb{Z}$ is the floor function. Some empirical checks confirm that increasing B with respect to the value above causes barely noticeable improvements (if any), both in terms of the calibration of significance levels (as long as the nominal value is not extremely small) and of power.

Nevertheless, if the goal was to control the FDR, the situation would be quite different — both Benjamini and Hochberg (1995) and Benjamini and Yekutieli (2001) are based on ordering *p*-values and, consequently, B cannot be too small with respect to the number of hypotheses that are tested, in order to avoid an excessive build-up of null *p*-values. Unfortunately, when it comes to analyzing real data, this becomes unfeasible (see the empirical issues in the upcoming Appendix E). Instead, we opted for controlling the FWER (and not the FDR), which has the slight drawback of neglecting the weak dependencies among the $\binom{L}{2}$ hypothesis tests (both due to each individual SNP being present in multiple pairs and due to linkage disequilibrium).

E Computational challenge of the resampling approach

The implementation of the brute-force testing procedure, as presented in Appendix D, was an extremely challenging issue from the computational point of view, given the high dimensionality of the data and the large number of hypothesis tests resulting from the combinatorial explosion. Thus, a quite sophisticated set of computer technologies and strategies was required to obtain results in somewhat manageable computational times.

Table S2 Comparison of running times for the different versions of the code, all of them referring to the permutation testing approach. It should be noted that the times for runs of the sequential code on the largest GWAS are estimations.

Code version	Simulation, $R = 10^3$	GWAS, $L = 1000$	GWAS, $L = 4000$
R sequential	12 h 10 min	42 days 1 h	2 years
R & C sequential	3 h 59 min	2 days 1 h	30 days
R & C parallel	50 min	2 h 41 min	2 days

As a general rule, any statistical technique based on GWAS data will suffer from the issues that are inherent to such input (high dimension and low sample size). To illustrate this point, Table S2 compares the run times of the original *R* code with another one, whose core is implemented in the compiled language *C*, this way making the numerical crunching far swifter. This second code —labeled “R & C” in the table— also includes some high-performance computing (HPC) improvements and, what is more, it can run in

© 2026 WILEY-VCH Verlag GmbH & Co. KGaA, Weinheim

sequential or in parallel mode (i.e., the workload can be distributed among different processors, increasing the computational speed by a factor that is approximately equal to the number of available processors). For a comparison of performance like the one in Table S2, it is crucial to carry on the experiments in the same environment, which in our case was supercomputer *Finisterrae II* (Galician Supercomputing Center, CESGA).

Hence, in light of the order of magnitude of these times (the *R* version would need up to two years in large-scale settings, while the *R* & *C* parallel implementation only requires ten hours), it is fully justified to resort to HPC strategies in a compiled language, especially if one takes into account that a GWAS can easily involve millions of SNPs, with the running time being a linear and monotonically increasing function of $\binom{L}{2}$ and, consequently, $O(L^2)$; as illustrated by the ratio between the GWAS columns of Table S2.

For the parallel version of the *R & C* code, in each case, the lowest amount of hardware that yielded results within a reasonable amount of time was used — 12 cores for simulations, and 48 processors for real data analyses. To reduce the times by a factor of f, it would suffice to increase the number of processors f times, as long as logistic constraints make it possible.

Moreover, the algorithm was parallelized in two alternative ways:

1. using a shared memory paradigm via the OpenMP library, distributing the computational effort among the different cores that exist within a processor;

2. applying a distributed memory strategy, where different computational nodes —that belong to various machines— are able to share workload via a message protocol, which in our case is the MPI library.

The first parallelization (that is very easy to implement in the main loop of the algorithm) was useful for applying the test to simulated data, where the dimensionality was not too problematic. However, the number of parallel running threads one can add is limited by the number of available cores in a CPU processor chip, and it is not enough to address real data examples. For this reason, a distributed memory parallelization was developed, with a classical leader/follower paradigm, where hundreds of processors can work together to reduce complexity. It consists in:

1. A processor (*leader*) calls *R* routines that load the matrices that contain the input, split it and distribute it among several processors (*followers*).

2. Each processor works with one fragment of the matrix, running the iterations that have been assigned to it (i.e., performing independence tests for a fraction of the total SNP pairs).

3. Once each follower finishes its part, it sends the results to the leader.

4. The leader builds the final *p*-value matrix, which is later used to wrap up the results in *R*.

Please note that “leader/follower” is just one of several proposed replacements for the historical name of this technology, which is linked to racial discrimination and we therefore avoid reproducing such nomenclature here.

The *R & C* version of our code combines an interface in the programming language *R* with a core in *C*, with the latter being devoted to performing low-level computations in a time-efficient manner. Another important factor that decreases running times in our implementation is the use of specific libraries to codify low-level operations that involve large vectors and matrices. Namely, the well-known Intel MKL libraries and SIMD (Single Instruction; Multiple Data) techniques have been applied to exploit data-level parallelism — using an extension in the registers and the arithmetic and logic instructions present in modern microprocessors, they can process the same operation simultaneously on the elements of an array through a single instruction. We found it particularly useful when it came to implementing matrix operations.

It was not possible to resort to preexisting software because the most efficient distance-correlation-related algorithms (like the one by Chaudhuri and Hu, 2019) are only designed for the Euclidean case and, therefore, not adaptable to the structure of the 3-point spaces that are the scope of our article.

© 2026 WILEY-VCH Verlag GmbH & Co. KGaA, Weinheim

F Genomic database

The SNP data used in our article come from a case-control study of schizophrenia, which was performed on $n_1 = 585$ patients and $n_2 = 573$ control blood donors, all of them of Galician origin, as described by Rodríguez-López *et al.* (2020).

Each individual's genome was sequenced using microarray *PsychArray-24 BeadChip* (Illumina, San Diego, California). After genotyping, several conventional quality controls were performed. Namely, to avoid experiment-derived problems, we excluded from the database every SNP that verified at least one of the following conditions:

1. The minor allele frequency (MAF) is less than 1 $\%$ in our samples.
2. The genotype proportions differ significantly from Hardy–Weinberg equilibrium in the control sample, for nominal $\alpha = 0.001$.
3. The *call rate* (proportion of non-missing data after genotyping) is under 95 $\%$, or it is significantly different between cases and controls ($p-$value of less than 0.001).

The previous filters aim at only retaining those SNPs for which one can clearly discriminate between the three possible genotypes.

Individuals were removed when, after the SNP quality control, the genotyping for more than 5 $\%$ of their SNPs was missing. For assessing cryptic relatedness, we computed the identity by descent proportion (pi-hat statistic) for each pair of individuals and, for every pair in which $\hat{\pi} > 0.15$, one of its members was removed.

All the aforementioned restrictions were applied with the default algorithms and implementations for GWAS quality controls of PLINK (Purcell et al., 2023).

The final SNP count for the schizophrenia database, which is used in Section 5 of the main article for illustrating our methodology, is $L = 6,371,078$. It contains observations of $n_1 = 585$ cases and $n_2 = 573$ controls.

Data collection followed the guidelines of the Declaration of Helsinki, was approved by the Galician Ethical Committee for Clinical Research, and participants signed an informed consent; as stated in Rodríguez-López *et al.* (2020).

G Technical details of the real data application

We now explain some non-essential technicalities that were left out of the explanation of the experimental procedure in Section 5 of our article.

G.1 Experiment I: Functional enrichment

For this experiment, among all the autosomal SNPs, only missense SNPs (i.e., those that induce a change in the amino acid sequence of a protein) were considered, as a way to detect interaction at the protein level.

To determine which SNPs are missense and which not, our reference was the ENSEMBL Biomart database (ENSEMBL, 2023). Since our schizophrenia data refer to the GRCh37.p13 (hg19) assembly of the human genome, we chose the Biomart version accordingly. For a comprehensive review of ENSEMBL and Biomart, we refer the reader to Kinsella *et al.* (2011).

As we wanted to have the ability to detect some signal, and it is remarkably difficult to detect any instance of epistasis in real data (Russ *et al.*, 2022), we restricted ourselves to those SNPs for which the least frequent of the two alleles is observed with a frequency of at least 0.05 in our samples and 0.01 at ENSEMBL Biomart. The latter filter also ensured that the missense SNPs under study are well known and annotated. This yielded a SNP count of $19,356$.

© 2026 WILEY-VCH Verlag GmbH & Co. KGaA, Weinheim

We then removed all the SNPs that lie in any of the 25 small regions of the human genome which are known to suffer from *long-range linkage disequilibrium* (LD), a phenomenon that can cause confusion between true SNP-SNP interactions related to schizophrenia and associations due to the architecture of chromosomes (Price *et al.*, 2008). The list of those 25 regions can be found, for example, in Facal *et al.* (2021).

Furthermore, once the high LD regions were removed, we followed standard practice among geneticists (Abdellaoui *et al.*, 2013) and, from the remaining $18,268$ SNPs, we *pruned* those showing evidence of short-range LD, setting $r^2 < 0.1$ in PLINK 1.9 (Purcell et al., 2023). We used windows of width 500 SNPs, shifting them $+1$ SNP at each step. Thus, we obtained the $L = 8030$ SNPs that were analyzed with our method in Experiment I of the main body of the article.

Whenever we had to assign SNPs to genes, we once again followed the annotations at ENSEMBL Biomart.

G.2 Experiment II: Gene expression

In Experiment II, we only used SNPs that regulate gene expression (i.e., eQTLs) in any of the brain tissues, and nowhere else in the human body, according to data from the GTEx Consortium (2023). With this aim, we downloaded `GTEx_Analysis_v7_eQTL.tar.gz` (single tissue cis-eQTL data for GTEx Analysis V7, dbGAP accession phs000424.v7.p2) from

`https://www.gtexportal.org/home/datasets`,

which again refers to the GRCh37.p13 (hg19) assembly of the human genome. There we simply removed anything that is not a SNP (e.g., insertions), created lists of SNPs that regulate gene expression in brain and non-brain tissues, and performed a set difference.

We therefore chose those SNPs present in our study which are also among the $97,913$ SNPs that act as eQTLs only in brain (and not in other tissues) according to GTEx. Removing data from sexual chromosomes, as well as the high LD regions (as in Experiment I), the SNP count dropped to $56,395$. After once again pruning the short-range LD, we got the final SNP list for this experiment, of length $L = 6456$.

References

Abdellaoui, A., Hottenga, J., de Knijff, P., Nivard, M., Xiao, X., Scheet, P. et al. (2013). Population structure, migration, and diversifying selection in the Netherlands. *European Journal of Human Genetics* **21,** 1277–1285.

Arcones, M. Á. and Giné, E. (1992). On the bootstrap of U and V-statistics. *Annals of Statistics* **20,** 655–674.

Benjamini , Y. and Hochberg, Y. (1995). Controlling the false discovery rate: A practical and powerful approach to multiple testing. *Journal of the Royal Statistical Society (Series B)* **57,** 289–300.

Benjamini , Y. and Yekutieli, D. (2001). The control of the false discovery rate in multiple testing under dependency. *Annals of Statistics* **29,** 1165–1188.

Cai, T. T. (2017). Global testing and large-scale multiple testing for high-dimensional covariance structures. *Annual Review of Statistics and Its Application* **4,** 423–446.

Cai, T. T. and Liu, W. (2016). Large-scale multiple testing of correlations. *Journal of the American Statistical Association* **111,** 229–240.

Chaudhuri, A. and Hu, W. (2019). A fast algorithm for computing distance correlation. *Computational Statistics & Data Analysis* **135,** 15–24.

Edelmann, D. and Goeman, J. J. (2022). A regression perspective on generalized distance covariance and the Hilbert–Schmidt independence criterion. *Statistical Science* **37,** 562–579.

Edelmann, D., Welchowski, T. and Benner, A. (2021). A consistent version of distance covariance for right-censored survival data and its application in hypothesis testing. *Biometrics* **78,** 867–879.

Emily, M. (2012). IndOR: A new statistical procedure to test for SNP-SNP epistasis in genome-wide association studies. *Statistics in Medicine* **31,** 2359–2373.

© 2026 WILEY-VCH Verlag GmbH & Co. KGaA, Weinheim

ENSEMBL (2023). ENSEMBL Biomart. `https://grch37.ensembl.org/biomart`.

Facal, F., Flórez, G., Blanco, V., Rodríguez, J., Pereiro, C., Fernández, J. M. et al. (2021). Genetic predisposition to alcohol dependence: The combined role of polygenic risk to general psychopathology and to high alcohol consumption. *Drug and Alcohol Dependence* **221,** 108556.

Frånberg, M., Gertow, K., Hamsten, A., PROCARDIS consortium, Lagergren, J., and Sennblad, B. (2015). Discovering genetic interactions in large-scale association studies by stage-wise likelihood ratio tests . *PLoS Genetics* **11,** e1005502.

Goudey, B., Rawlinson, D., Wang, Q., Shi, F., Ferra, H., Campbell, R. M. et al. (2013). GWIS — Model-free, fast and exhaustive search for epistatic interactions in case-control GWAS. *BMC Genomics* **14**.

GTEx Consortium (2023). The Genotype-Tissue Expression Project. `https://www.gtexportal.org` .

Gusareva, E. S. and Van Steen, K. (2014). Practical aspects of genome-wide association interaction analysis. *Human Genetics* **133,** 1343–1358.

Gyenesei, A., Moody, J., Semple, C. A. M., Haley, C. S. and Wei, W.-H. (2012). High-throughput analysis of epistasis in genome-wide association studies with BiForce. *Bioinformatics* **28,** 1957–1964.

Hemani, G., Theocharidis, A., Wei, W.-H. and Haley, C. S. (2011). EpiGPU: Exhaustive pairwise epistasis scans parallelized on consumer level graphics cards. *Bioinformatics* **27,** 1462–1465.

Henry, V. J., Bandrowski, A. E., Pepin, A.-S., González, B. J. and Desfeux, A. (2014). OMICtools: An informative directory for multi-omic data. *Database* **2014,** bau069.

Huang, C. and Huo, X. (2022). A statistically and numerically efficient independence test based on random projections and distance covariance. *Frontiers in Applied Mathematics and Statistics* **7,** 779841.

Jakobsen, M. E. (2017). Distance covariance in metric spaces: Non-parametric independence testing in metric spaces. `https://arxiv.org/abs/1706.03490v1`.

Kam-Thong, T., Czamara, D., Tsuda, K., Borgwardt, K., Lewis, C. M., Erhardt-Lehmann, A. et al. (2011). EPI-BLASTER —fast exhaustive two-locus epistasis detection strategy using graphical processing units. *European Journal of Human Genetics* **19,** 465–471.

Kam-Thong, T., Azencott, C.-A., Cayton, L., Pütz, B., Altmann, A., Karbalai, N. et al. (2012). GLIDE: GPU-Based linear regression for detection of epistasis. *Human Heredity,* 73, 220–236.

Kinsella, R., Kähäri, A., Haider, S., Zamora, J., Proctor, G., Spudich, G. et al. (2011). ENSEMBL BioMarts: A hub for data retrieval across taxonomic space. *Database* **2011**.

Moore, J. H. and Hill, D. P. (2015). Epistasis analysis using artificial intelligence. *Methods in Molecular Biology* **1253,** 327–346.

Niel, C., Sinoquet, C., Dina, C. and Rocheleau, G. (2015). A survey about methods dedicated to epistasis detection. *Frontiers in Genetics* **6**.

Pecanka, J., Jonker, M. A., International Parkinson's Disease Genomics Consortium, Bochdanovits, Z. and van der Vaart, A. W. (2017). A powerful and efficient two-stage method for detecting gene-to-gene interactions in GWAS. *Biostatistics* **18,** 477–494.

Ponte-Fernández, C., González-Domínguez, J. and Martín, M.J. (2022). Fiúncho: A program for any-order epistasis detection in CPU clusters. *Journal of Supercomputing* **78,** 15338–15357.

Price, A., Weale, M., Patterson, N., Myers, S., Need, A., Shianna, K. et al. (2008). Long-range LD can confound genome scans in admixed populations. *American Journal of Human Genetics* **83,** 132–135.

Purcell, S. et al. (2023). PLINK v1.9: Whole genome association analysis toolset. `https://www.cog-genomics.org/plink` .

Ritchie, M. D., Hahn, L. W., Roodi, N., Bailey, L. R., Dupont, W. D., Parl, F. F. and Moore, J. H. (2001). Multifactor-dimensionality reduction reveals high-order interactions among estrogen-metabolism genes in sporadic breast cancer. *American Journal of Human Genetics* **69,** 138–147.

Rodríguez-López, J., Arrojo, M., Paz, E., Páramo, M. and Costas, J. (2020). Identification of relevant hub genes for early intervention at gene coexpression modules with altered predicted expression in schizophrenia. *Progress in Neuro-Psychopharmacology and Biological Psychiatry* **98,** 109815.

Russ, D., Williams, J., Cardoso, V., Bravo-Merodio, L., Pendleton, S., Aziz, F., Acharjee, A. and Gkoutos, G. (2022). Evaluating the detection ability of a range of epistasis detection methods on simulated data for pure and impure epistatic models. *PLoS One* **17,** e0263390.

© 2026 WILEY-VCH Verlag GmbH & Co. KGaA, Weinheim

Schwarz, D. F., König, I. R. and Ziegler, A. (2010). On safari to random jungle: A fast implementation of random forests for high-dimensional data. *Bioinformatics* **26,** 1752–1758.

Shang, J., Sun, Y., Liu, J. X., Xia, J., Zhang, J. and Zheng, C.-H. (2016). CINOEDV: A co-information-based method for detecting and visualizing $n-$order epistatic interactions. *BMC Bioinformatics* **17,** 214.

Sun, R., Xia, X., Chong, K. C., Zee, B. C.-Y., Wu, W. K. K. and Wang, M. H. (2019). Wtest: An integrated R package for genetic epistasis testing. *BMC Medical Genomics* **12,** 180.

Sun, Y., Shang, J., Liu, J.-X., Li, S. and Zheng, C.-H. (2017). EpiACO – a method for identifying epistasis based on ant colony optimization algorithm. *BioData Mining* **10**.

Székely, G. J. and Rizzo, M. L. (2017). The energy of data. *Annual Review of Statistics and Its Application* **4,** 447–479.

Székely, G. J., Rizzo, M. L. and Bakirov, N. (2007). Measuring and testing dependence by correlation of distances. *Annals of Statistics* **35,** 2769–2794.

Wan, X., Yang, C., Yang, Q., Xue, H., Fan, X., Tang, N. L. S. and Yu, W. (2010a). BOOST: A fast approach to detecting gene-gene interactions in genome-wide case-control studies. *American Journal of Human Genetics* **87,** 325–340.

Wan, X., Yang, C., Yang, Q., Xue, H., Tang, N. L. S. and Yu, W. (2010b). Predictive rule inference for epistatic interaction detection in genome-wide association studies. *Bioinformatics* **26,** 30–37.

Wang, Y., Liu, X., Robbins, K. and Rekaya, R. (2010). AntEpiSeeker: Detecting epistatic interactions for case-control studies using a two-stage ant colony optimization algorithm. *BMC Research Notes* **3,** 117.

Zhang, Y. and Liu, J. S. (2007). Bayesian inference of epistatic interactions in case-control studies. *Nature Genetics* **39,** 1167–1173.

© 2026 WILEY-VCH Verlag GmbH & Co. KGaA, Weinheim
