# Supplementary material for: Testing for Genetic Interactions in Complex Disease With Distance Correlation
Source: Biom J. 2026 Sep 7;68(5):e70150. doi: 10.1002/bimj.70150 (PMC13548348; doi:10.1002/bimj.70150)
Supplement: Supplementary file 2 [file BIMJ-68-e70150-s001.zip › simulation_study/Power_indep_qexp_BOOST.pdf]

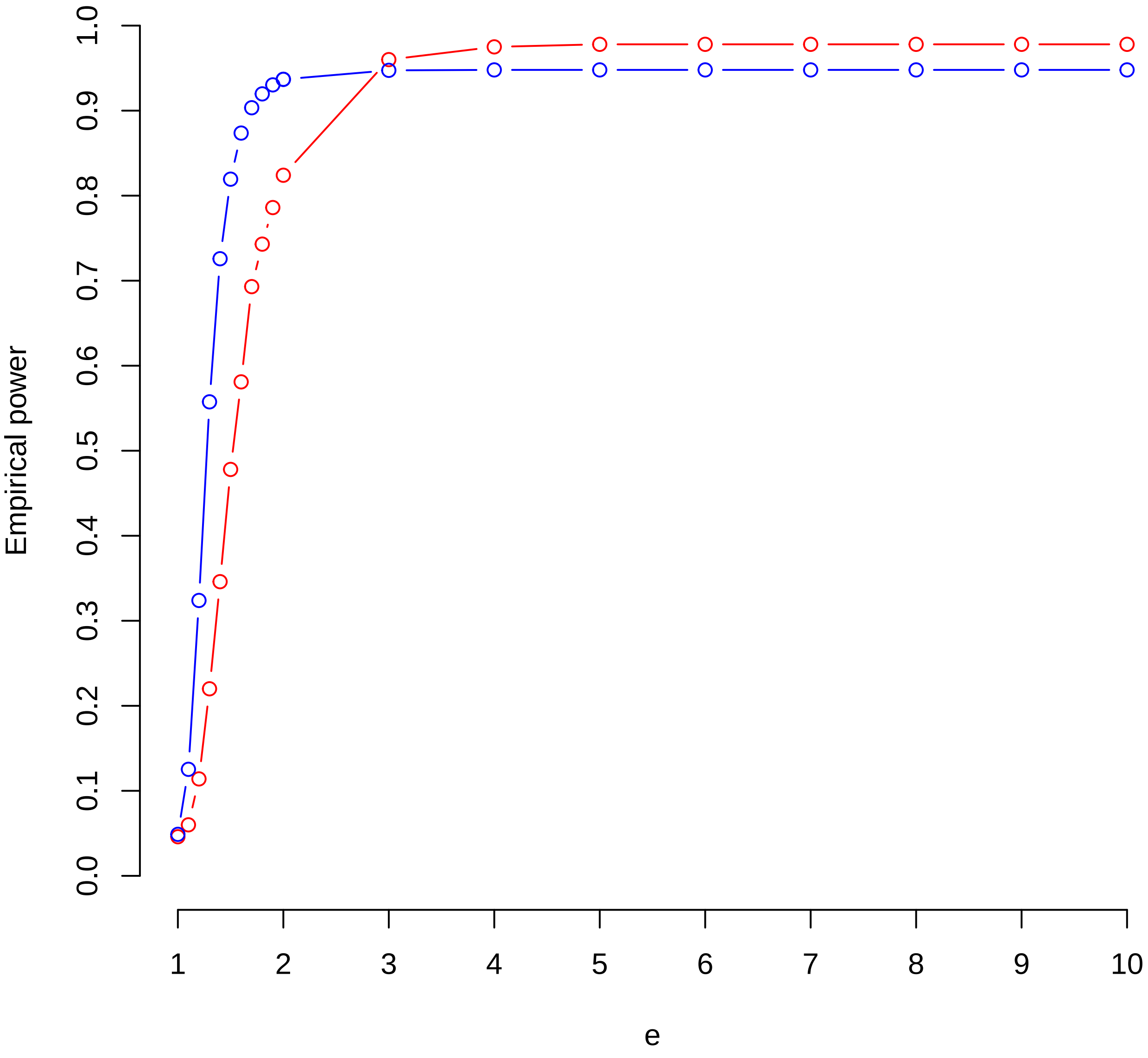

Empirical power
0.0
0.1
0.2
0.3
0.4
0.5
0.6
0.7
0.8
0.9
1.0
1
2
3
4
5
6
7
8
9
10
e
