# Supplementary material for: Testing for Genetic Interactions in Complex Disease With Distance Correlation
Source: Biom J. 2026 Sep 7;68(5):e70150. doi: 10.1002/bimj.70150 (PMC13548348; doi:10.1002/bimj.70150)
Supplement: Supplementary file 2 [file BIMJ-68-e70150-s001.zip › README.pdf]

Title: Testing for genetic interactions in complex disease with distance correlation

Authors: Fernando Castro-Prado, Javier Costas, Dominic Edelmann,
Wenceslao Gonzalez-Manteiga, and David R. Penas

Corresponding author for code: Fernando Castro-Prado. **Email:** f.castro.prado *at* pm *dot* me

Configurations:
After running the session info command, we get the following information on our R session.

```
> sessionInfo()
R version 4.3.1 (2023-06-16 ucrt)
Platform: x86_64-w64-mingw32/x64 (64-bit)
Running under: Windows 10 x64 (build 19045)

Matrix products: default

locale:
[1] LC_COLLATE=Spanish_Spain.utf8  LC_CTYPE=Spanish_Spain.utf8
[3] LC_MONETARY=Spanish_Spain.utf8 LC_NUMERIC=C
[5] LC_TIME=Spanish_Spain.utf8

time zone: Europe/Madrid
tzcode source: internal

attached base packages:
[1] stats     graphics  grDevices utils     datasets  methods   base

other attached packages:
[1] ggplot2_3.4.3    momentchi2_0.1.5 coga_1.2.0

loaded via a namespace (and not attached):
 [1] utf8_1.2.3       R6_2.5.1         magrittr_2.0.3   gtable_0.3.4     glue_1.6.2
 [6] tibble_3.2.1     pkgconfig_2.0.3  lifecycle_1.0.3  cli_3.6.1        fansi_1.0.5
[11] scales_1.2.1     grid_4.3.1       vctrs_0.6.3      withr_2.5.1      compiler_4.3.1
[16] tools_4.3.1      pillar_1.9.0     munsell_0.5.0    Rcpp_1.0.11      colorspace_2.1-0
[21] rlang_1.1.1
```

General information
The rest of the present README file explains how to reproduce the simulations (calibration of the type I error and power comparison with BOOST) and the real data analyses (Experiments I and II) in our manuscript. For each of these tasks one will find the necessary files in a subfolder with a straightforward name within the *Code_and_Data* folder.

In order to run the code more quickly, we provide an extended version of the code and data folder, which can be downloaded from Zenodo: https://doi.org/10.5281/zenodo.20744952 . That larger folder contains a number of very large matrices with intermediate results, which make it possible to just reproduce chunks of the code or specific numerical examples in our article.

Reproducing our simulation study
By simply running the R script *masterscript_power.R*, one generates the data tables (as **.dat* files) necessary to produce the plots on Section 4 of the manuscript. The power plots (which include the comparison with competing method BOOST, in a different colour) are directly generated when running the masterscript. The code for the calibration plots is a bit more cumbersome, due to the confidence band, so we split it to a separate script. Just run *plotting_calibration.R* to obtain Figure 2 on the main body of the manuscript.

The masterscript is provided within the subdirectory */simulation_study/* of the main *Code_and_Data* folder, and so is the aforementioned script for producing the calibration plots. The */simulation_study/* subfolder also contains two R files (*power_simulation_functions.R* and *aux_functions_boost.R*) with

auxiliary functions that are used by the main scripts, the <u>*plink.exe*</u> file (to run BOOST), PDF samples of the plots that are expected to be obtained with each re-run, and a crucial empty folder (named <u>*/fi/*</u>), where dump files for the runs involving BOOST are stored. The code is written expecting the existence of <u>*/fi/*</u>, so it is important not to delete it (even when disposing all its content).

Reproducing the illustration for a pair of SNPs
Obtaining the results in Section 5.1 (illustration of our method for a specific pair of SNPs) is quite straightforward, once the exact coordinates of the SNPs under consideration are given. To this purpose, we provide a very short script, called <u>*illustration_npsr1_gnas.R*</u>, within folder *experiment_i*. Running it yields the two *p*-values reported in that section.

The other numerical result in 5.1 the combined confidence score of the functional interaction between proteins NPSR1 and GNAS. In order to reproduce it, one should visit www.string-db.org, introduce manually "NPSR1\n GNAS" in the "Multiple proteins" text box, select *Homo sapiens* as the target species, press "Continue" and look for the "Exports" section in the results page. All this refers to version 12 of the STRING database, and this is a screenshot of the results page (which may look different once STRING is updated in the future):

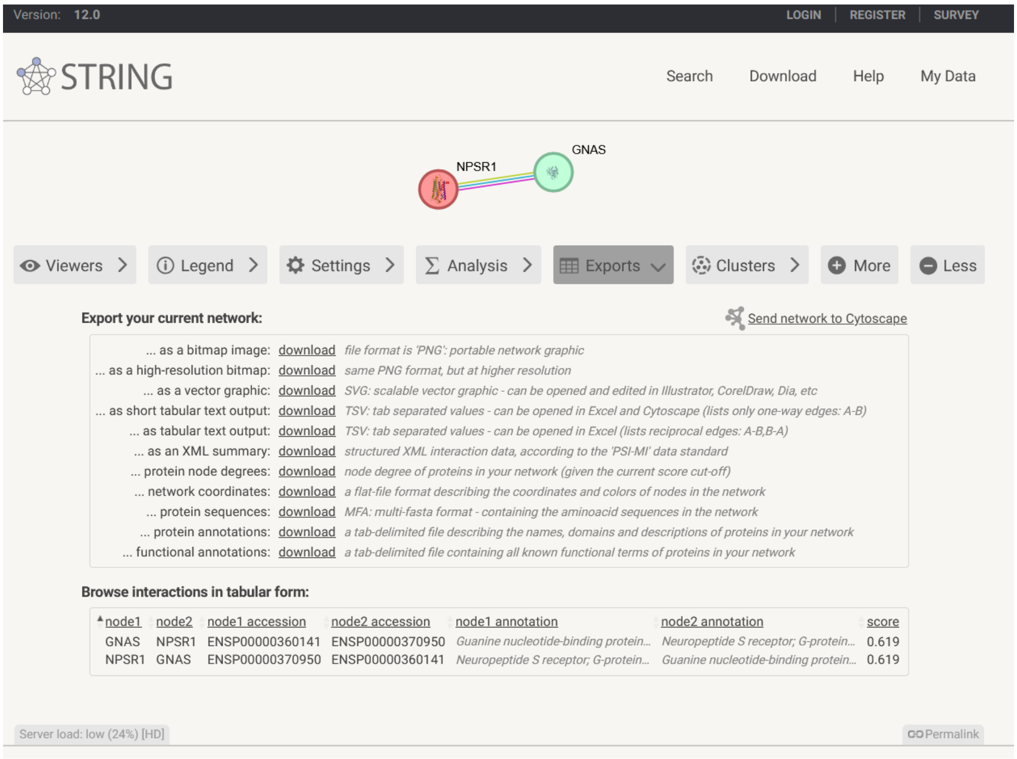

Reproducing Experiment I
We use our full schizophrenia GWAS database, as described in appendix F of our supplement. We assume that we have a triplet of PLINK files (**.bed*, **.bim*, **.fam*) within the *experiment_i* folder. We are not allowed to share ours, but one can run the script <u>*filtering_snps_experiment_i.R*</u> to obtain the matrices with the observations for cases and controls with the same filters that we describe in the supplement. In our case, this yields L=8030 SNPs for Experiment I. We indicate as comments of the **.R* file the results of each relevant step.

One should run <u>*masterscript_experiment_i.R*</u> to reproduce Experiment I. It uses as input the data from the 8030 SNPs for cases and controls (*Matrix_X.dat* and *Matrix_Y.dat*), as well as the SNP IDs in the chromosome-position format (with some alterations for the sake of anonymity of sampled individuals, in order to make this data shareable; such modifications do not influence the results we present). The latter can be found in *chr_pos.dat*. Every relevant result has been written down as a comment in the **.R* file. We recommend using the search function with the query “*result present in the manuscript*” to find the exact lines of code that replicate every numerical result for Experiment I that is cited in the main manuscript.

At many points of the script, we generate **.dat* files with intermediate results, in order to allow running only parts of it. We do this in light of the moderately long running times of some segments, but it is also feasible to run the entire script within reasonable time in any modern personal computer. When running only segments of this script or the one for Experiment II, attention should be paid to the fact that some

variables in both scripts share the same name (and so do part of the *.*dat* files). It is recommended to start a new R session when skipping from one experiment to the other, or at least to clean the R environment.

Please note that it is necessary to set as the working directory the location of the masterscript R file before running it.

Reproducing Experiment II

As in Experiment I, we begin with the full GWAS database. We assume that we have a triplet of PLINK files (**.bed, *.bim, *.fam*) within the *experiment_ii* folder. We are not allowed to share ours, but one can run the script *filtering_snps_experiment_ii.R* to obtain the matrices with the observations for cases and controls with the same filters that we describe in the supplement. In our case, this yields L=6456 SNPs for Experiment II. We indicate in the comments of the **.R* file the results of the relevant steps.

An important caveat is that we do not attach the GTEx files necessary for running this script. For obtaining them, one should visit the GTEx Portal on https://www.gtexportal.org/home/datasets , download the file *GTEx_Analysis_v7_eQTL.tar.gz* (single tissue cis-eQTL data for GTEx Analysis V7, dbGAP accession phs000424.v7.p2), unzip it and place all the *Brain_*.signifpairs.txt* files within the folder *Code_and_Data/experiment_ii/gtex_v7_signifpairs/brain* , and all the remaining **.signifpairs.txt* files (the ones not beginning with *Brain_**) in the analogous *nonbrain* folder.

To replicate Experiment II, the reader is kindly asked to run *masterscript_experiment_ii.R*. All the observations we made for the masterscript of Experiment I also apply to this one.

Source of data

The origin and nature of our schizophrenia database is described in appendix F of the supplement (see also the acknowledgment section on the main document, for the funding institutions that were involved in the data generation process).

Due to confidentiality, we are not allowed to share the whole database, so we will directly start the reproducibility scripts with the two subsets that are used on Experiment I (8030 SNPs) and Experiment II (6456) SNPs. We do provide scripts with the necessary code for filtering GWAS datasets in the same way we did in each experiment (file names *filtering_snps_experiment_i[i].R*)

Please note that some SNP IDs have been altered for the purpose of sharing our code for reproducibility, in a way that preserves anonymity of sampled individuals and maintains the results we present.
